# Analysing human neural stem cell ontogeny by consecutive isolation of Notch active neural progenitors

**DOI:** 10.1038/ncomms7500

**Published:** 2015-03-23

**Authors:** Reuven Edri, Yakey Yaffe, Michael J. Ziller, Naresh Mutukula, Rotem Volkman, Eyal David, Jasmine Jacob-Hirsch, Hagar Malcov, Carmit Levy, Gideon Rechavi, Irit Gat-Viks, Alexander Meissner, Yechiel Elkabetz

**Affiliations:** 1 Department of Cell and Developmental Biology, Sackler School of Medicine, Tel Aviv University, Tel Aviv 69978, Israel; 2 Broad Institute of MIT and Harvard, Cambridge, Massachusetts 02142, USA; 3 Department of Stem Cell and Regenerative Biology, Harvard University, Cambridge, Massachusetts 02138, USA; 4 Department of Cell Research and Immunology, Faculty of Life Sciences, Tel Aviv University, Tel Aviv 69978, Israel; 5 Cancer Research Center, Chaim Sheba Medical Center, Tel Hashomer 52621, Israel; 6 Sackler School of Medicine, Tel Aviv University, Tel Aviv 69978, Israel; 7 Department of Human Molecular Genetics and Biochemistry, Sackler School of Medicine, Tel Aviv University, Tel Aviv 69978, Israel

## Abstract

Decoding heterogeneity of pluripotent stem cell (PSC)-derived neural progeny is fundamental for revealing the origin of diverse progenitors, for defining their lineages, and for identifying fate determinants driving transition through distinct potencies. Here we have prospectively isolated consecutively appearing PSC-derived primary progenitors based on their Notch activation state. We first isolate early neuroepithelial cells and show their broad Notch-dependent developmental and proliferative potential. Neuroepithelial cells further yield successive Notch-dependent functional primary progenitors, from early and midneurogenic radial glia and their derived basal progenitors, to gliogenic radial glia and adult-like neural progenitors, together recapitulating hallmarks of neural stem cell (NSC) ontogeny. Gene expression profiling reveals dynamic stage-specific transcriptional patterns that may link development of distinct progenitor identities through Notch activation. Our observations provide a platform for characterization and manipulation of distinct progenitor cell types amenable for developing streamlined neural lineage specification paradigms for modelling development in health and disease.

## Abstract (web summary)

Profiling pluripotent stem cell (PSC)-derived neural progeny is of fundamental interest for characterizing stem cell differentiation. Here, the authors analyse neural progenitors consecutively derived from human PSCs, showing dynamic stage-specific transcriptional patterns for distinct neural progenitors.

## Graphical abstract

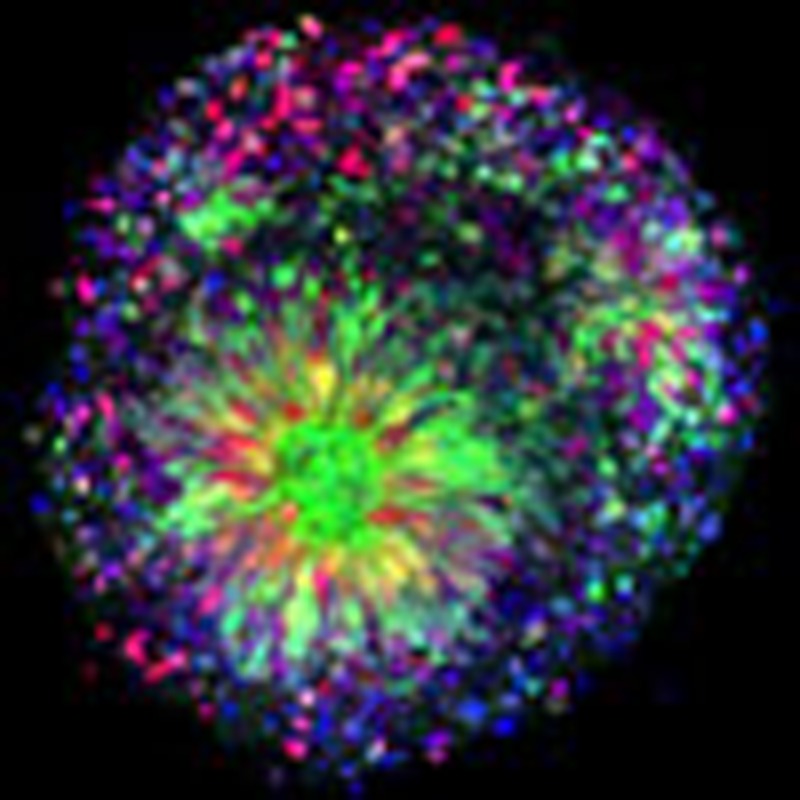

The identification of NSCs in the developing and adult brain has transformed the way we understand central nervous system (CNS) development and regeneration. However, long following their isolation from the CNS^1^ or the derivation of neural progenitors from PSCs, our ability to address the dynamic changes in self-renewal and potency of distinct NSC types *in vitro* has remained poor. The exceptionally pioneering studies done in the NSC field *in vivo* have led to the identification of fundamental NSC types populating the germinal zones—neuroepithelial (NE) cells, radial glial (RG) cells and adult NSCs (aNSCs; for review see refs 2, 3). These studies provided the basis for our understanding of the dynamic nature and lineage relationship of these distinct NSC types *in vivo*, describing the unique timing mechanism of neuronal cell type generation^4^. However, in depth *in vitro* dissection of the molecular characteristics of each stage, particularly within the RG compartment, has been stalled mainly by the heterogeneity of NSC cultures and the lack of stage-specific markers. In fact, despite being highly heterogeneous^5^, distinct RG cell types as well as aNSCs are known to share similar RG cell markers rather than distinctive ones. The reporter gene- and surface marker-based prospective isolation of acute mouse aNSCs serves as a great example for a more in depth analysis of aNSC characteristics^6^. However, applying such a study to human CNS-derived RG cells is limited due to obvious shortage in early human CNS tissue. Thus, in depth understanding on human NSC ontogeny and dynamics in culture is still elusive.

The advent of PSCs has brought the ability to direct early neural progenitors towards a range of neuronal cell fates including midbrain dopaminergic neurons^7^, spinal motoneurons^8^ and telencephalic cortical neurons^9^,^10^,^11^ (for review see ref. 12). One remarkable study by Knoblich and coworkers allows monitoring early to midgestation cerebral morphogenesis and neurogenesis, making up an attractive approach to model development and disease of the human brain^13^. Another recently published comprehensive work delineates the temporal transcriptome analysis of cerebral cortex neuronal subtypes derived from PSCs^14^. These two latter advancements have significantly helped to demonstrate the capability of hESC differentiation strategies to recapitulate major hallmarks of *in vivo* neural development and serve as a valuable resource for modelling development and disease of the human brain. Further to these important findings, however, there is a need to better understand how different types of progenitors emerge and exert their full potential while progressing through distinct competences during development. Addressing such an aim requires employing differentiation culture strategies that allow distinguishing primary progenitor cells holding extensive proliferation capacity and broad differentiation potential from the bulk of accompanying progenitors that lack these abilities. We previously isolated an early progenitor cell type from PSCs that exhibits considerable self-renewal capacity (termed rosette-neural stem cells (R-NSCs)), and showed their developmental potential and distinct molecular signature^15^. However, also the R-NSC stage exhibits high heterogeneity with respect to NSC potential and corresponds to a transient stage *in vitro*. Currently there is no list of genes at high confidence that are known for specific types of neural progenitors emerging in culture, stressing the need to unravel generalized networks and pathways involved in the extensively changing dynamics of early NE cells. Taken together, despite many years of NSC research, the heterogeneity and rapid transition through distinct neural stem and progenitor cell types still impedes our understanding of origin, lineage transitions and the key factors that maintain or alter the epigenetic stability of early NE cells.

To begin to tackle these fundamental challenges, here we establish a long-term neural differentiation system from PSCs using *HES5::eGFP* reporter human embryonic stem cell (hESC) line. HES5 is a major and direct downstream target of Notch activation pathway (for review see ref. 16). This allows the prospective isolation and characterization of primary progenitors retaining low proneural transcriptional activity and broad developmental potential and thus serving as the primary progenitors—or NSCs—that generate neural cellular diversity. The stepwise isolation of Notch active NSCs during neural differentiation of PSCs enables a systematic investigation of human NSC ontogeny and proposes a controlled module-based platform for understanding the development of normal and pathogenic NSCs and their progeny.

## Results

### Notch activation links major neural lineage transitions

**Figure 1:**
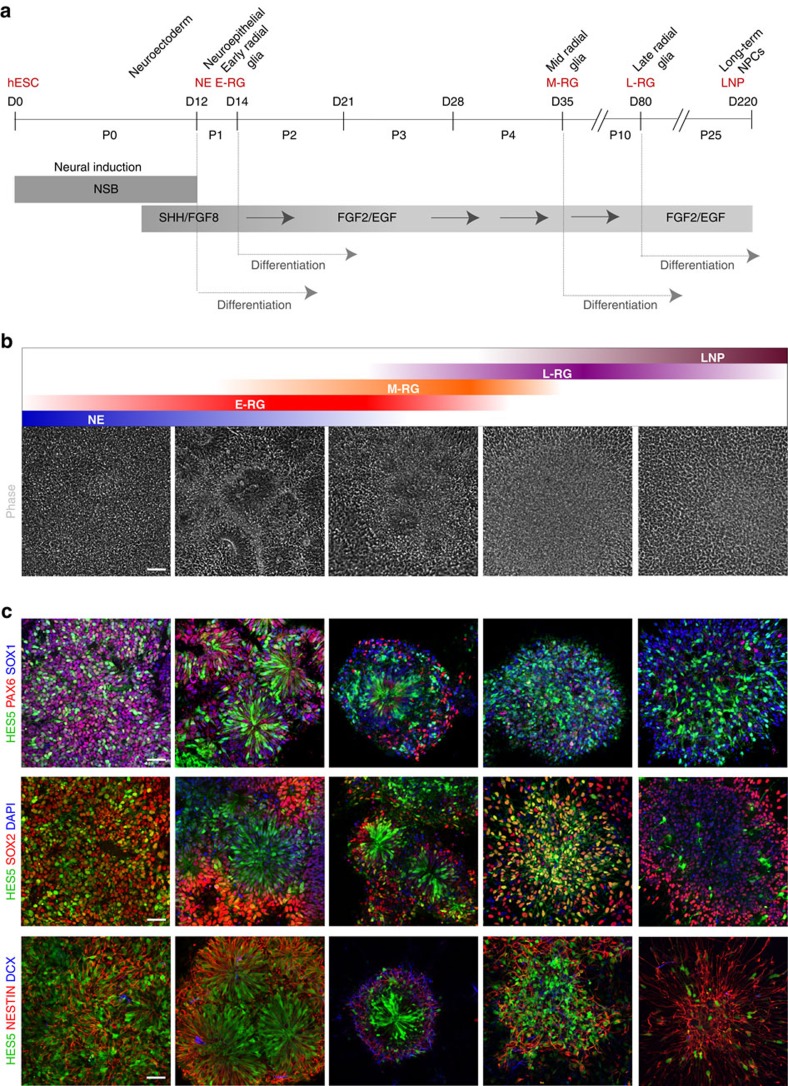
Notch activation links major neural lineage transitions in hESC derived neural progenitor cells. (**a**) Neural differentiation scheme. Neural induction was performed by a dual SMAD inhibition protocol followed by long-term propagation with the factors indicated for 220 days. Naming conventions representing neuroepithelial (NE), early radial glial (E-RG), midradial glial (M-RG), late radial glial (L-RG) and long-term cultured progenitors (LNP) are indicated. Number of passages are indicated as P(n). (**b**) Bright field microscopy of progenitor cells during long-term differentiation shows dynamic morphological features. Scale bar: 50 μm (valid for all images in **b**). (GFP matched images can be seen in Supplementary Fig. 1a). (**c**) Combined *HES5::eGFP* reporter expression and Immunostainings of stem/progenitor cell as well as differentiation markers throughout the progression period. Top: PAX6, SOX1 and HES5 induction during early stages (See also Supplementary Fig. 1a,b for *HES5::eGFP* percentages). Middle and Bottom: SOX2, NESTIN and DCX expression. Scale bar: 50 μm (valid for all images in **c**). Individual qPCR analyses for all genes tested at all stages are shown in Supplementary Fig. 1d.

We used the previously established H9 (WA09) derived *HES5::eGFP* hESC reporter line^17^ to monitor morphology and HES5 reporter cell expression dynamics. We defined five consecutive stages during 220 days of neural differentiation and propagation (Fig. 1a,b; Supplementary Fig. 1a,b). Neuroectodermal cells emerged as early as day 5–8 and expressed SOX1 followed by PAX6, but not HES5 (Supplementary Fig. 1c). On day 12, HES5 is widely expressed and coincides PAX6 and SOX1, along with other progenitor cell markers such as SOX2 and NESTIN (Fig. 1c; Supplementary Fig. 1d), possibly marking establishment of the CNS earliest NE cells following neural induction^18^. Shortly after, on day 14, HES5-expressing cells rapidly become elongated, maintain PAX6 expression and form neural rosettes—highly polarized structures containing radially organized columnar cells^15^—reminiscent of RG cells residing within the developing ventricular zone (VZ)^19^,^20^ and as suggested by other *in vitro* studies^9^,^10^,^11^. Neural rosettes last till approximately day 35 (Fig. 1b,c; Supplementary Fig. 1a). We therefore designated day-14 rosettes as early radial glial (E-RG) cells and day-35 rosettes as midradial glial (M-RG) cells. HES5 continues to be expressed in progenitors throughout the progression period, albeit in progressively decreasing numbers (Supplementary Fig. 1b). In contrast, SOX1, SOX2 and NESTIN remained highly expressed in the majority of cells throughout the entire propagation (Fig. 1c). This indicates that the highly proliferative conditions are not sufficient to retain the initial high Notch activation level beyond the E-RG stage. More importantly, this may reflect the transition of early NSCs into more limited progenitors, in line with *in vivo* findings^21^,^22^. This observation was also accompanied by an apparent expression of DCX at the M-RG and L-RG stages, together with a gradual loss in rosette integrity (Fig. 1c). Taken together, these findings suggest that extensive neurogenesis occurs mainly during M-RG through L-RG stages. On the basis of these observations we defined two additional post rosette consecutive stages for analysis—day 80 and day 220. Neural progenitors on day 80 represent a later radial glial (L-RG) cell population exhibiting a more gliogenic bias, based on downregulation of rosette (R-NSC) markers such as PLZF and the upregulation of glial markers such as epidermal growth factor receptor (EGFR) and S100B^15^ (Supplementary Fig. 1e). These were still capable of generating neurons and glia, supporting existence of subsets of NSCs^15^. Neural progenitors could continuously propagate for many additional passages. Day 220 represents a long-term cultured neural progenitor (LNP) stage exhibiting a further substantial increase in EGFR and S100B levels (Supplementary Fig. 1e), while retaining multipotency (Supplementary Fig. 1f). These dynamic changes in Notch activation state along with morphological features suggest that this long-term culture system provides a suitable paradigm to study NSC state and cell fate transition.

### Notch activation confers CNS rostrocaudal patterning ability

**Figure 2:**
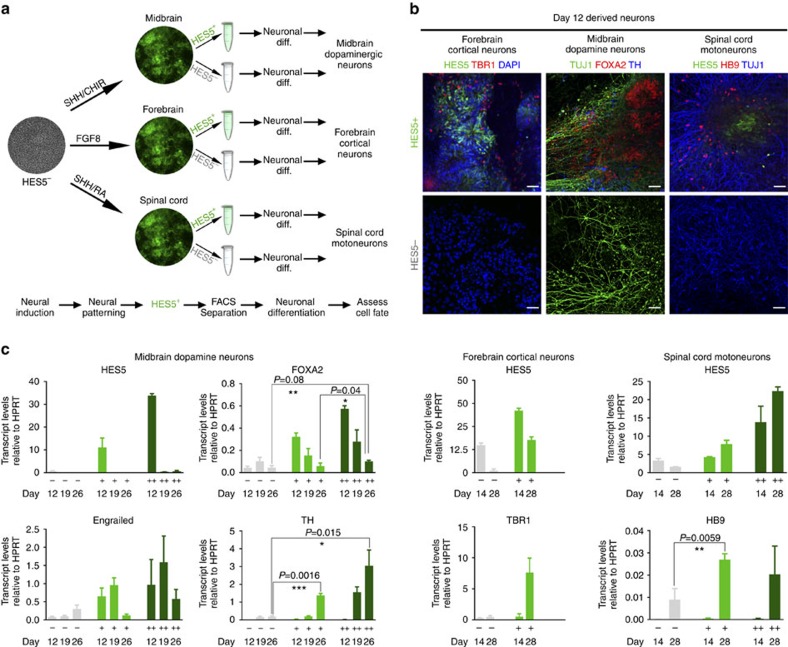
Early Notch activation in NE cells confers amenability to neural patterning cues. (**a**) Neural patterning paradigm scheme. PSCs were subjected to neural induction and were exposed to patterning cues directing differentiation into forebrain, midbrain and spinal cord cell fates with the morphogenes indicated. Region specific progenitors were sorted to high, medium or low *HES5::eGFP* expressing populations followed by neuronal differentiation. (**b**) Immunostaining for respective neuronal progeny derived from HES5+ (top) or HES5− (bottom) isolated on day 12 of progression. Cortical neurons marked by TBR1, midbrain dopaminergic neurons marked by FOXA2/TH and spinal cord motoneurons marked by HB9 are shown. Scale bar: 50 μm. (**c**) Quantitative PCR analysis of transcript levels of HES5 as well as selected regional markers in high (++, dark green bars), medium (+, light green bars) and low (−, grey bars) HES5-expressing progenitors, in their proliferative state (day 12 or day 14) and following terminal neuronal differentiation (day 19, day 26, or day 28). All transcript levels shown are normalized to respective HPRT levels in each sample. Values were obtained from three technical replicates. Statistical analysis: mean±s.e.m.; *t*-Test: ***P<0.001; **P<0.01; *P<0.05. Individual qPCR analyses for additional regional or neuronal markers are shown in Supplementary Fig. 2a.

To dissect the early cell fate potential of HES5+ compared with that of HES5− isolated progenitor cell populations, we tested whether early Notch activation is required for NE cells to respond to early developmental cues that yield regionally specified CNS neurons. We exposed neuroectodermal cells to patterning cues directing rostrocaudal regional fates before onset of *HES5::eGFP* expression. When neuroectodermal cells reached the NE stage (day 12), HES5+ and HES5− cells were separated, further subjected to complete differentiation along the selected regional paradigm, and were finally assessed for their ability to yield the corresponding regional-specific neuronal subtypes (Fig. 2a). Remarkably, early-projection neurons expressing appropriate rostral to caudal regional neuronal markers such as TBR1 forebrain cortical neurons, FOXA2/TH midbrain dopaminergic neurons and HB9 spinal motoneurons, could be generated mainly from high HES5-expressing cells (Fig. 2b,c). In contrast, HES5− progenitors weakly responded to patterning cues although they were capable of generating neurons (Fig. 2b, bottom; Supplementary Fig. 2a). Requirement for Notch activation in the generation of early CNS neurons was also evident for additional early cortical neuronal markers such as CTIP2, NR2F1 and PCP4 (ref. 23) (Supplementary Figs 4 and 5). Finally, we also confirmed requirement for Notch activation by inhibiting this signalling pathway using DAPT during neural induction. Both HES5 and PAX6 expression levels were reduced following DAPT addition, while the neural crest/placodal marker SIX1 was upregulated (Supplementary Fig. 2b).

These findings suggest that neuroectodermal cells require high Notch activation to acquire appropriate CNS neuronal cell identities. To further support this latter possibility, we followed HES5+ and HES5− progenitors derived from the NE stage through the E-RG stage and assessed their cell fate and proliferative capacities with respect to Notch activation. We found that consecutively sorted HES5+ populations retained PAX6 expression, while consecutively sorted HES5− cells retained AP2A expression, confirming that CNS and neural crest fates are dictated by Notch active and inactive states, respectively (Supplementary Fig. 3a). Furthermore, additional CNS markers such as SOX2 and OTX2 were enriched in HES5+ cells at the NE stage compared with HES5− cells, while the neuronal marker DCX was enriched in HES5− cells (Supplementary Fig. 3b). Finally, consecutively sorted HES5+ populations retained an overall stable level of BrdU incorporation, compared with consecutively sorted HES5− cells (Supplementary Fig. 3c). These results suggest that Notch in neuroectodermal cells is mainly important for segregating CNS from non-CNS cell fates and in addition may confer CNS cells with a proliferative advantage.

### Notch activation enables cortical lamination and glial fates

Studies on cortical differentiation from PSCs have shown how continued culture of early rosettes yields sequentially appearing cortical neuronal layers by a default intrinsic mechanism^9^,^10^,^11^. Here we asked whether HES5+ NE cells generated by such a default model serve as the primary progenitor cell source also for cortical lamination. We specifically asked whether prospective purification of Notch active progenitor cells throughout the progression *in vitro* correlates with potential to yield cortical neurons in a time and cortical layer dependent manner.

**Figure 3:**
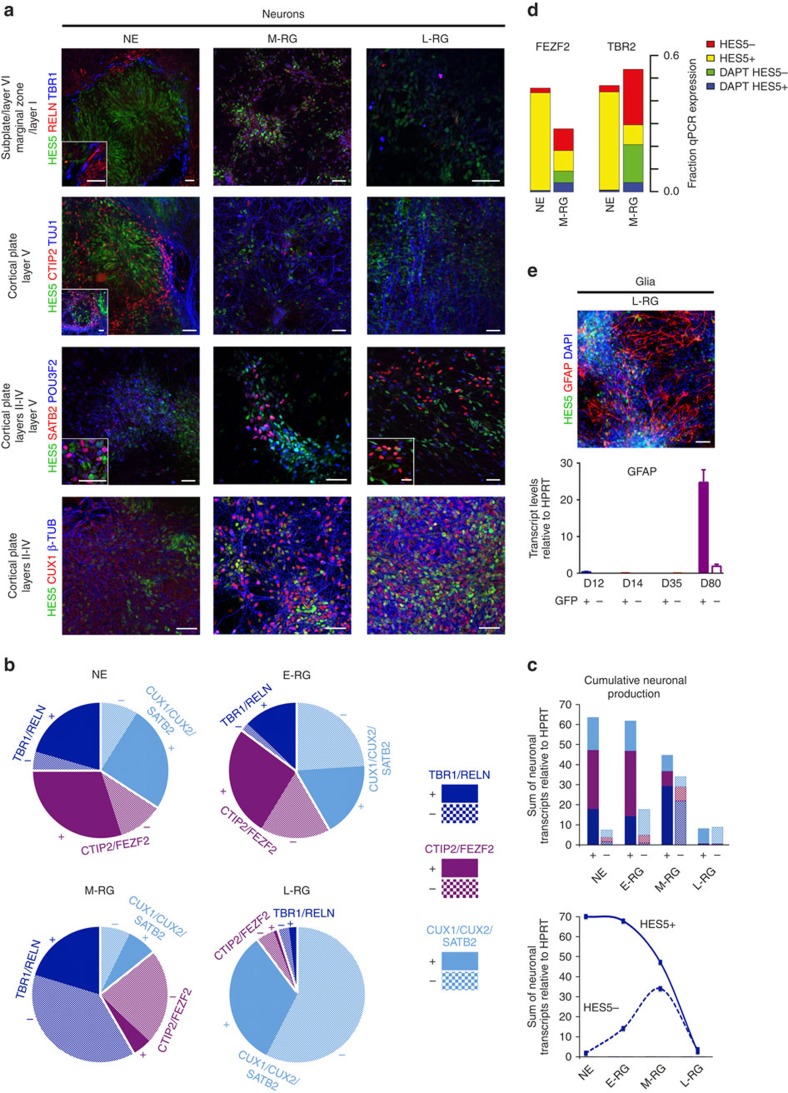
Consecutive isolation of Notch active progenitors recapitulates cortical lamination and glial fates. (**a**) Combined *HES5::eGFP* reporter expression and immunostainings of cortical layer specific neuronal markers: Early born neurons expressing TBR1, RELN and CTIP2 (top two panels), and late derived neurons expressing SATB2, POU3F2 and CUX1 (bottom two panels), are shown for NE, M-RG and L-RG progenitors that were subjected to neuronal differentiation. Insets for RELN/TBR1 and SATB2/POU3F2 show magnified areas within the image. Inset for CTIP/TUJ1 shows same magnification but a different view of neuronal axons. Images of HES5+ derived neurons are shown. Scale bars: 50 μm for images, 25 μm for Insets. Images of HES5− derived neurons and percentages of all cortical subtypes derived from both HES5+ and HES5− cells are presented in Supplementary Fig. 5. (**b**) Distribution of relative transcript abundance based on qPCR for selected stage-specific marker gene groups for either deep or upper layer neuronal progeny. Contributions of HES5+ and HES5− populations per each respective stage are shown. Marker gene groups for each progenitor stage were created by collapsing the normalized values of TBR1/RELN, CTIP2/FEZF2 and CUX1/CUX2/SATB2 (see Methods for details). Individual qPCR analyses for all genes tested at all stages are shown in Supplementary Fig. 4. (**c**) Same as in **b**. Here, cumulative neuronal marker levels based on relative transcript levels are shown (top). Note the decrease in total neuronal progeny shown in the lower panel, as the glial marker GFAP is upregulated in panel **e**. (**d**) Distribution of relative transcript abundance based on qPCR for selected stage-specific marker genes for indicated progenitor or neuronal cell markers. Contributions of HES5+ and HES5− populations per each respective stage from either untreated or DAPT treated cells are shown. Expression levels relative to HPRT of all four conditions (colour coded) were summed per each gene and plotted as a single bar. (**e**) Top: Combined *HES5::eGFP* reporter expression and immunostaining of the glial marker GFAP following differentiation of the L-RG stage. Scale bar: 50 μm. Bottom: GFAP transcript level for distinct progenitor stages as assessed by qPCR. Values were obtained from three technical replicates. Statistical analysis: mean±s.e.m.

We found that early NE and E-RG progenitor stages gave rise mainly to neurons populating deep layers (Fig. 3a) and in a Notch-dependent manner (Fig. 3b,c; Supplementary Figs 4 and 5). These included deep layer-V FEZF2+ and CTIP2+ corticospinal neurons^24^, early subplate and deep layer-VI TBR1+ corticothalamic neurons^25^, early marginal zone RELN+ Cajal Retzius neurons^26^, and deep layer SATB2+ callosal neurons. While the latter are mainly known in their contribution to upper layers, they have been also shown to reside within deep layers to some extent^27^. In contrast, later M-RG and L-RG stages gave rise mainly to neurons that populate superficial layers (Fig. 3a) and in a Notch-independent manner (Fig. 3b,c; Supplementary Figs 4 and 5). These included CUX1+ and CUX2+ (ref. 28) as well as SATB2+ layers II-IV callosal neurons. We interestingly noticed that while CUX1/2 protein levels were induced in neurons derived from M-RG progenitors and onwards, at the RNA level they were induced already at early-stage-derived neurons, and this transcript expression depended on Notch activation (Fig. 3b,c; Supplementary Fig. 4). This suggested that early CUX1/2 RNA expression reflected early progenitor potential, rather than immediate competence, to generate superficial layer neurons. This result is paralleled by an *in vivo* observation according to which progenitors prospectively labelled for *Cux2* appear already during early cortical development^29^. Taken together, these results show that early progenitor stages require Notch activation to generate early appearing neurons, while late progenitor stages yield later derived neurons regardless of Notch activation.

We hypothesized that M-RG stage progenitors did not require Notch activation for generating later derived neurons because many of them correspond to HES5− subventricular zone (SVZ)-like intermediate progenitor (INP) cells that have already accumulated from earlier stages in a Notch-dependent manner. To test this, we asked whether the generation of such SVZ progenitors expressing TBR2 (EOMES)^30^ requires Notch activation. We found that TBR2 was upregulated during differentiation of NE cells in a Notch-dependent manner and that this upregulation was prevented following Notch inhibition by DAPT (Fig. 3d). This is in contrast to the later (M-RG) stage, where most TBR2 levels were derived from HES5− cells, and accordingly were not inhibited by DAPT. This shows that the majority of TBR2 progenitors that were apparent at the M-RG stage were already generated from early Notch active cells rather than generated *de novo* at the M-RG stage. For comparison, we also tested the expression of FEZF2—a hallmark of earliest cortical RG progenitors^31^,^32^. FEZF2 expression at early stages also strictly depended on Notch activation and was fully inhibited by DAPT (Fig. 3d). The inhibition of FEZF2 and TBR2 by DAPT demonstrates that generation of both early and late progenitors and their neurons is significantly affected in the absence of Notch activation.

Additional support for stage-specific differential dependence on Notch is provided by TBR1+ and RELN+ neurons. These appear not only during early sublate and marginal zone formation, respectively, but also during midgestation by later SVZ progenitors^33^, and may also populate more caudal cortical regions^34^. Accordingly, we found that TBR1 and RELN neurons could be both generated also at the M-RG stage and in a Notch-independent manner (Fig. 3b,c; Supplementary Figs 4 and 5).

Similar to the critical role of Notch activation during the derivation of early progenitor cells and their neuronal progeny, also the generation of astrocytes expressing GFAP at the L-RG stage required Notch activation (Fig. 3e). This is in contrast to neurons at that stage, which could be derived also from HES5− cells (Fig. 3b,c). Taken together, these results show that the distinct progenitor units spatiotemporally organized in the developing VZ and SVZ and which are responsible for cortical lamination and glial transformation *in vivo*, can be consecutively isolated from PSCs through sustained Notch signalling *in vitro*. While the main role of Notch activation is to promote the perpetuation of potent progenitors through culture, it may not be directly involved in generating cellular diversity, but rather maintain progenitors responsive to our culture conditions, which instruct these cell fate changes.

### Notch activation links hallmarks of cortical development

**Figure 4:**
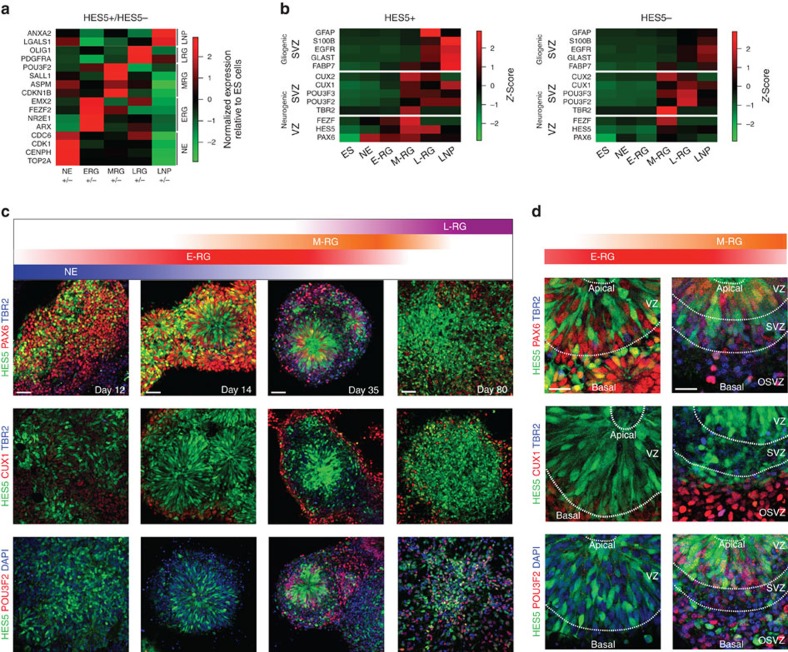
Transition through progenitor cell stages demarcates developing rosettes as VZ and SVZ equivalents. (**a**) Differential expression levels for selected genes that are most differentially expressed between HES5+ and HES5− cells in a stage-specific manner. Selected gene members are indicated on the left, developmental stages are indicated on the bottom, and gene categories classified by stage are indicated on the right. Values plotted on the heatmap represent ratios of expression levels relative to ES cells. (**b**) Relative expression levels (*z*-scores) based on microarray expression data for the entire differentiation time course for selected germinal zone marker genes. Relative expression levels are shown for HES5+ (top) and HES5− (bottom) samples separately. Genes are ordered from VZ to SVZ and from neurogenic to gliogenic markers. Individual qPCR analyses for all genes tested at all stages are shown in Supplementary Fig. 6c. Note that the apparently high GFAP expression in HES5+ cells at the L-RG stage has in fact low absolute expression values, and only appear high relatively to expression in other stages (all stages per each gene are normalized to 1; that is, highest red intensity). To compare GFAP transcript levels during proliferation and serum induced astrocytic differentiation, see Figs 5d and 3e, respectively. (**c**) Combined *HES5::eGFP* reporter expression and immunostainings of neural stem/progenitor markers, RG markers, and proliferation markers throughout the progression period. From top: PAX6 marking the VZ and TBR2 marking the SVZ are shown. Middle: CUX1 marking SVZ is shown. Bottom: the (mainly) SVZ marker POU3F2 is shown. Scale bar: 50 μm (valid for all images in **c**). (**d**) High-power magnification of E-RG and M-RG images shown in **c**. Dashed lines demarcate proposed VZ, SVZ and OSVZ regions, containing apical RG, INPs and basal RG, respectively. Scale bar: 25 μm (valid for all images in **d**).

We next looked into the molecular hallmarks that specifically define each of the developmental stages *in vitro* with respect to Notch activation. We employed global gene expression analysis (Supplementary Data 1; see Methods for details) and specifically investigated transcripts differentially expressed in HES5+ compared with HES5− progenitors at each stage (Fig. 4a; Supplementary Data 2). Interestingly, genes upregulated in HES5+ NE cells were mainly associated with cell cycle progression and DNA replication, and included *CDC6*, *CDK1*, *CENPH* and *TOP2A* (Fig. 4a). Genes specifically enriched in HES5− cells at the NE stage included the proneural genes *NEUROD4*, *NGN1/2*, *TBR2* and *DCX* (Supplementary Data 3). These results are compatible with NE HES5+ acting as symmetrically dividing NSCs during early nervous system development (for review see ref. 21) and further suggest that Notch confers amenability for regional neuronal specification through promoting cell cycle.

In contrast to NE cells, HES5+ at the E-RG stage (day 14) were enriched for genes such as *ARX, FEZF2 and NR2E1* with respect to HES5− cells (Fig. 4a), indicating that Notch active NE cells underwent a sharp and rapid transition towards an RG cell stage with a strong dorsocaudal telencephalic character. Notch active progenitors in the more advanced rosette M-RG stage continued to highlight cerebral developmental genes such as *POU3F2 (BRN2)* as well as genes associated with neuroblast cell division such as *ASPM* (Fig. 4a), fitting with extensive neurogenesis during the M-RG stage. Transcripts overrepresented in HES5+ cells in the L-RG stage were associated with glial fate key genes such as *OLIG1* and *PDGFRA* (Fig. 4a). Finally, genes overrepresented in HES5+ versus HES5− progenitors at the LNP stage such as *ANXA2* and *LGALS1 (GALECTIN)* are associated with ependymal cells and aNSCs^6^ (Fig. 4a), suggesting that LNP cells progressed beyond RG fates towards adult-like progenitor identity. These results show that PSC-derived neural progenitors progress through distinct stages that may be possibly linked via Notch activation, from NE cell proliferation through neurogenic RG cell differentiation, glial transformation and adult NSC specification. To further support these observations we looked specifically at the relative distribution of germinal zone genes among the various stages with respect to Notch activation. The heatmap in Fig. 4b (see also Supplementary Fig. 6c) shows a consecutive correlation of NE/E-RG, M-RG, L-RG and LNP stages *in vitro*, with VZ, early SVZ, late SVZ and subependymal zone (SEZ) *in vivo*, respectively. Furthermore, it is evident that VZ markers are enriched in Notch active cells while SVZ markers are more comparably distributed between Notch active and inactive cells. To summarize, our molecular data further confirm that Notch activation links the establishment of NE cells and their transition through consecutive primary RG progenitors.

### Early- and midrosettes demarcate germinal zone equivalents

We next employed immunostainings and 3D (three-dimensional) reconstruction analyses to dissect the hierarchical progression of progenitors at the cellular and cytoarchitectural levels with respect to Notch signalling. The abundant occupancy of PAX6 and HES5 at all rosette cells at the E-RG stage (Fig. 4c,d) indeed fits the dorsal cortex molecular identity of E-RG cells (Fig. 4a)^10^,^11^. In contrast, PAX6 and HES5 spatial distribution in the M-RG stage was mainly confined to lumens, as well as to regions located distally to rosette areas (Fig. 4c,d). These two PAX6 and HES5-expressing progenitor cell types possibly corresponded to VZ residing apical RG progenitors and putative outer SVZ (OSVZ) localized basal RG progenitors, respectively^35^,^36^. 3D reconstruction analysis of E-RG and M-RG rosettes demonstrates that HES5+ cells are composed of elongated radial fibres that cross the entire Z-section in an apical to basal manner, attesting for a complex rather than flat rosette composition obtained via non-confocal images. E-RG rosettes (Supplementary Movie 1) are packed with HES5+/PAX6+ cells across all rosette area while dividing nuclei located luminally, at multiple Z-levels. M-RG rosettes are characterized by HES5+/PAX6+ cells and dividing nuclei both confined to luminal regions only, at multiple Z-levels, in addition to neuronal processes accumulating at lower Z-levels (Supplementary Movie 2). The cell division at luminal sites is also reflected by the expression pattern of the M-phase marker PHH3, which is confined to nuclei within lumens at E-RG and M-RG rosettes, while the general cell cycle marker KI67 was apparent among all progenitors regardless of HES5 expression (Supplementary Fig. 6a).

Further evidence ascribing the M-RG stage rosettes as a site of midneurogenesis is also provided by the many TBR2+ INPs that appeared transiently and specifically at this stage, and were located at rosette peripheries, assigning these regions in M-RG rosettes as midneurogenesis SVZ-like areas (Fig. 4c,d; Supplementary Fig. 6b)^30^,^33^. This was further corroborated by the expression of CUX1/2 and POU3F2/3. These neuronal markers begin to be expressed in VZ/SVZ progenitors during midgestation^28^,^37^, and accordingly appeared at the M-RG stage, located at rosette peripheries (Fig. 4c,d; Supplementary Fig. 6b).

### Rosette disassembly marks the beginning of gliogenesis

**Figure 5:**
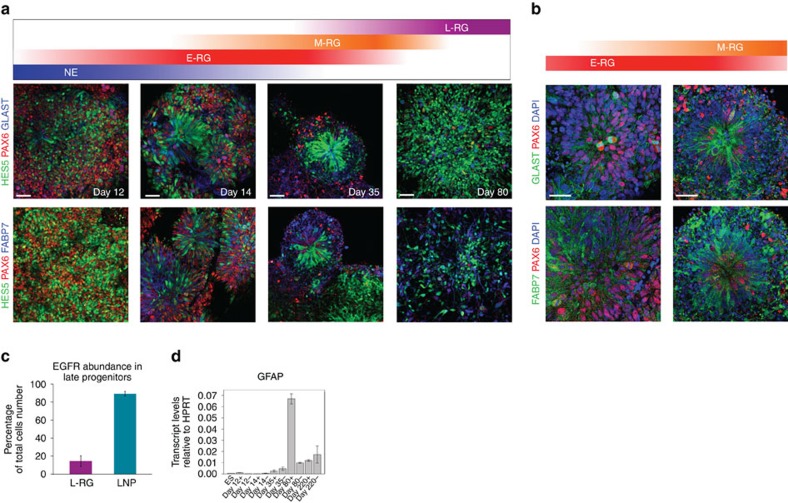
Glial transformation with respect to Notch activation. (**a**) Combined *HES5::eGFP* reporter expression and immunostainings of the RG markers GLAST (top) and FABP7 (bottom). Scale bar: 50 μm (valid for all images in **a**). (**b**) High-power magnification of E-RG and M-RG images for selected genes shown in **a**. Scale bar: 25 μm (valid for all images in **b**). (**c**) EGFR expression percentages by FACS analysis for L-RG (purple) and LNP (turquoise) stages is shown. Average of 2 independent experiments is shown. Statistical analysis: mean±s.e.m. (**d**) Relative GFAP expression levels based on qPCR data for the entire progression period. Relative expression levels are shown for HES5+ and HES5− samples during progenitor proliferation. Values were obtained from three technical replicates. Statistical analysis: mean±s.e.m. Compare the very low relative levels of GFAP during proliferation (day 80 HES5+ cells) to GFAP levels at the same progenitor type following astrocytic differentiation in Fig. 3e.

Evidence suggesting that M-RG rosettes serve not only as a site of extensive neurogenesis, but also of transition to glial stages is provided by the expression pattern of the RG markers GLAST and FABP7. These became evident already in E-RG rosettes, coinciding with PAX6 and HES5 (Fig. 5a,b), marking neurogenic RG. At the M-RG stage these markers appeared at luminal regions together with HES5 and PAX6, but were also located at rosettes peripheries where they did not coexpress PAX6 and HES5. This fits the findings that CNS progenitors prospectively tagged for GLAST and FABP7 at early stages *in vivo* were found labelling most neuronal progeny, while if prospectively marked at midneurogenesis, they labelled glial fates^38^.

The L-RG and LNP stages were no longer capable of forming rosettes, reflecting loss of epithelial integrity due to accumulation of basal progenitors, neurons and cells with astroglial character. HES5 and PAX6 cells further decreased in numbers (Fig. 1c; Supplementary Fig. 1b), reflective of the reduction in neurogenic NSCs. Some CUX1/2 and POU3F2 progenitors still remained at the L-RG stage, marking residual neurogenesis (Fig. 4c,d). Enhanced astroglial identity is supported by the further increase in GLAST and FABP7 levels (Fig. 5a,b) as well as the glial markers S100B and EGFR (Supplementary Fig. 1e). The increase in EGFR transcript levels was also reflected by an increase in EGFR+ cells in 10% of L-RG cells, as judged by fluorescent-activated cell sorting (FACS) analysis (Fig. 5c). EGFR labelling possibly reflected a newly established subset of progenitors at the L-RG stage, compatible with EGFR labelling mainly late SVZ progenitors *in vivo*^39^.

In contrast to L-RG cells, most LNP cells expressed EGFR as shown by FACS analysis (Fig. 5c), suggesting that following long-term culture, progenitors correspond to EGFR+ transit amplifying cells. Such cells can be derived from aNSC astrocytes following activation by EGF in culture^40^. This observation may explain the low GFAP levels found in our cultures throughout the progression period (Fig. 5d). Interestingly, many HES5-expressing cells at the LNP stage colocalized with S100B (Supplementary Fig. 1e), indicative of ependymal cells^41^, and in line with enrichment of genes harbouring ependymal character in HES5+ LNP cells (Fig. 4a).

### Molecular characterization of neural cell fate transition

**Figure 6:**
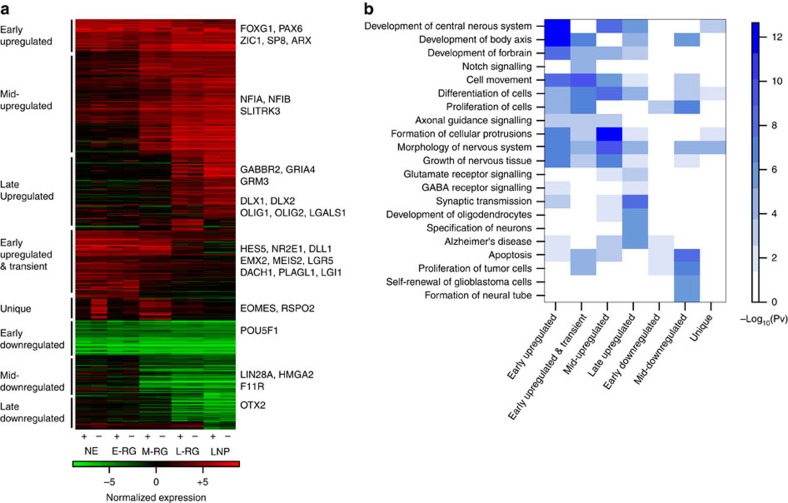
Global gene expression cluster analysis for stage specifically expressed genes. (**a**) Selected trends of global gene expression clustering comprising 496 genes using *k*-means (*k*=100) for all differentially expressed genes across the differentiation time course of HES5+ cells. Gene expression levels were log_2_ transformed and normalized to hESC by subtracting their respective hESC level. For all stages expression levels in HES5+ and HES5− cells are shown. Selected gene members of each cluster are indicated on the right while the cluster naming conventions are indicated on the left. Arrays were obtained from single replicates first used as a discovery tool, and then extensively validated by qPCR and immunostainings from independent experiments throughout the manuscript. (**b**) Gene set enrichment analysis results (using IPA, P-values are calculated using right-tailed Fisher exact test) of gene sets selected from the top 10 categories for each cluster are shown. Colour code indicates −log_10_ *P*-value.

Our findings suggest that HES5 expression during progenitor progression links the sequential transition through distinct competences. Such a mechanism can underlie the generation of heterogeneity in culture due to the fact that many HES5− cells exist throughout culture. Accordingly, factors that share expression among HES5+ cell stages may serve as transcriptional regulators for neural development, while stage-specifically expressed factors may be coopted to drive the transition through distinct competences. To identify such potential candidate genes having a role in inducing, maintaining, or transitioning between distinct competences, we employed an unbiased clustering analysis on all differentially expressed genes across HES5+ and HES5− populations. This analysis yielded 26 gene clusters that were divided to 7 distinct gene expression patterns (Fig. 6a; Supplementary Data 4). Gene clusters upregulated early from ES to NE cells and sustained among all stages are expected to have a role in inducing neural fates and maintain anterior cell character throughout prolonged periods. Accordingly, this early upregulated cluster is enriched for central nervous system (*P*=8E−13; right-tailed Fisher exact test used by IPA) and forebrain (*P*=1.0E−8) development as well as neuronal cell movement (*P*=1.0E−10) GO categories (Fig. 6b), and contained factors such as *FOXG1*, *PAX6*, *ZIC1* and *SP8*, which have been well implicated either in neural induction, forebrain specification and cortical areal patterning. Gene clusters upregulated at the M-RG stage and sustained throughout subsequent stages are anticipated to have role in active neurogenesis but also in the initiation of gliogenic bias, in correlation with our findings (Figs 4 and 5). As such, this cluster was enriched for genes involved in morphology of nervous system (*P*=7.9E−7) and formation of plasma membrane projections (*P*=2.2E−6), both implicated in neuronal axon maturation. These categories included for example *NFIA* and *NFIB*, which are interestingly involved in both repressing neuronal progenitor state through Notch signalling concomitantly with activating glial fates^42^. Other factors included are *SLITRK*, *ASCL1* and *PREX1*, which are associated with neurite outgrowth as well as neuronal maturation and migration. In addition, we interestingly found that *EZH2*—the histone methyltransferase of PRC2, is transiently expressed through the M-RG stage (Supplementary Fig. 7a). This latter observation nicely correlates the finding that Ezh2 regulates the balance between self-renewal and differentiation in the mouse cerebral cortex, as its loss leads to aberrant timing of cortical development^43^. One particularly interesting cluster is characterized by genes exhibiting a transient expression during the NE stage (specifically in HES5− cells) followed by a transient reexpression during the M-RG stage. This cluster included *TBR2*, *RSPO2*, *NEUROD1* and *TFAP2B*—genes associated with neurogenesis and basal progenitor (INP) cell fate. This observation may intriguingly imply that the establishment of a set of transcription factors (TFs) regulating SVZ generation and cortical expansion may already originate and act during early corticogenesis. Genes upregulated at the L-RG and LNP stages are highly enriched for genes involved in neurotransmission (*P*=2.4E−7) and include *GABBR2*, *GRIA4* and *GRM3*, but are also enriched for genes strongly implicated in glial fates such as *OLIG1* and *OLIG2* (*P*=3.4E−7), again manifesting how late neuronal maturation events coincide with extensive gliogenesis. Another gene expressed at the LNP stage is *LGALS1* (Supplementary Fig. 7a,b). Interestingly, *Lgals1* was shown to be specifically enriched in prospectively isolated GFAP+/Prominin1+ aNSCs as well as ependymal cells^6^. Gene clusters upregulated at the NE towards E-RG stage were enriched for nervous system morphogenesis (*P*=1.2E−7) and cancer associated factors (*P*=6.9E−8) and included genes such as *NR2E1 and LGR5. NR2E1* is mainly expressed in Notch active cells at the E-RG stage (Supplementary Fig. 7a), compatible with *Nr2e1* role in controlling proliferation of VZ progenitors during the establishment and expansion of the SVZ^44^. Interestingly, NR2E1 was also moderately expressed at the later LNP stage (Supplementary Fig. 7a), in correlation with its expression in mouse aNSC astrocytes as well as its role also in brain tumor initiation from NSCs^45^. *LGR5*—another interesting E-RG specific gene (Supplementary Fig. 7a)—is a major stem cell regulator of adult tissue regeneration and malignancy, and was initially identified in the stem cells of the small intestine and colon^46^. Finally, we also identified a cluster of genes expressed in ES cells but also transiently in NE and E-RG stages. One such candidate is *LIN28A* (Supplementary Fig. 7b). Interestingly, this RNA-binding protein is known also to have a role in reprogramming to pluripotency^47^, suggesting additional roles for this protein during early neural development. Accompanying in this cluster is *HMGA2* (Fig. 6a)—a fetal and young-adult (but not old) NSC marker^48^ and a target for Let-7, a microRNA whose maturation and function is repressed by *LIN28A*. Finally, we also identified F11R (Supplementary Fig. 7b). This tight junction protein shown to be involved in platelet adhesion to the activated endothelium^49^, but was also suggested to act in the cell-to-cell adhesion of neuroepithelial cells^50^. Taken together, these transcriptional trends suggest that the dynamic changes occurring in progenitor cell potency during culture are linked via Notch activation through stage-specifically acting factors.

## Discussion

This study offers a first in depth dissection of the dynamic changes that lead to heterogeneity in PSC-derived neuroepithelial cells during long-term culture, and shows that they match developmental logics and timing principles of mammalian NSC ontogeny. Moreover, this study suggests that Notch activation is a critical component orchestrating this ontogeny *in vitro*, by establishing the identity of neuroepithelial cells, regulating their numbers during progression, and linking their transition through distinct developmentally specific primary progenitor cells—which together comprise the diversity of NSC types promoting neurogenesis and gliogenesis of the CNS.

**Figure 7:**
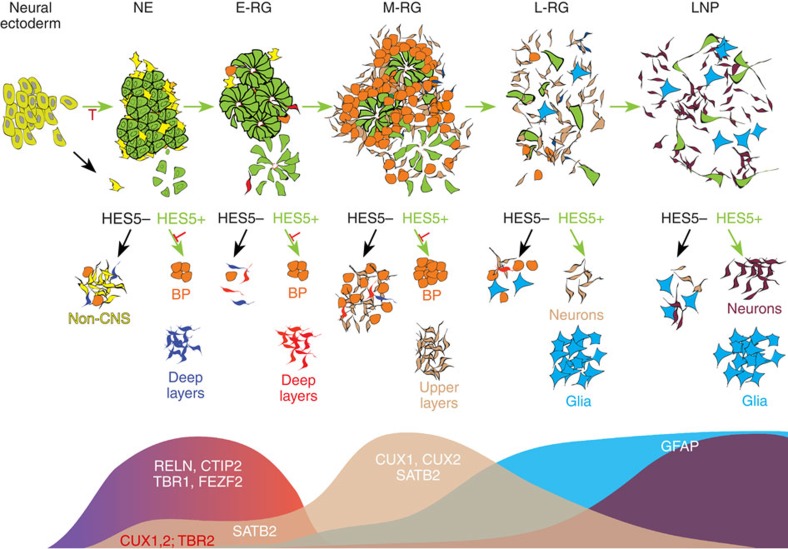
Schematic model for NSC progression. Neuroectodermal cells yield the earliest NE cells of the CNS by launching Notch activation and HES5 expression, while non-CNS neuroectodermal cells lack this activation. Under proliferation conditions, HES5+ NE cells yield consecutive radial glial progenitor cell types and their corresponding neuronal and glial progeny, hence considered as primary NSCs generating CNS neural diversity. Following mitogen withdrawal, HES5+ NE cells exert their competence towards deep-layer-specific neuronal types (RELN, TBR1 but also FEZF2 and CTIP2; blue-to-red wave, bottom panel) and do so in a Notch-dependent manner. In addition, they also upregulate SVZ progenitor markers such as TBR2 and CUX1, CUX2 at the RNA level (Red font, light brown early wave; bottom panel) and in a Notch-dependent manner, implying on their future potential to generate these progenitors at later stages. In contrast to NE cells, HES5+ E-RG cells are already committed to early dorsocaudal cortical identity, based on their elongated polarized cell morphology, rosette formation capacity and FEZF2 and EMX2 expression. Hence, they exhibit competence towards deep layer neurons (CTIP2, FEZF2; blue-to-red wave, bottom panel). M-RG stage cells are characterized by lower HES5 percentages, reduced rosette organization, substantial accumulation of HES5+-derived HES5− progenitors expressing CUX1, CUX2 and TBR2 at the protein level, and competence for yielding upper layer neuronal fates (CUX1, CUX2, SATB2; light brown second wave, bottom panel) in a Notch-independent manner. HES5+ L-RG cells are able to give rise to astrocytes in a Notch-dependent manner (GFAP; light blue wave, bottom panel), yet both HES5+ and HES5− cells at that stage continue to contribute to neurogenesis. Ultimately, L-RG cells transform to long-term progenitors (LNP) associated with adult NSC progeny (purple wave, bottom panel). Horizontal green arrows mark transition in a Notch-dependent manner. Diagonal green and black arrows mark HES5+ and HES5− cells, respectively, subjected to differentiation following FACS-based separation. When indicated, Notch active pathways were confirmed by DAPT (red bar-headed lines). Top panel shows cell types and developmental potential. Bottom panel shows temporal phases of neuronal and glial markers derived by the stages indicated above. BP, basal progenitors.

The consecutive prospective isolation of Notch active progenitors along the entire differentiation period *in vitro* enabled us to enrich cultures for primary progenitor cells that may hold proliferative advantage and broad developmental potential, while eliminating those lacking these features. This allowed the generation of distinct progenitor modules *in vitro* temporally linked via Notch activation to serve as building blocks of nervous system establishment and neocortical construction (see Model, Fig. 7). It is conceivable that each of the distinct HES5+ populations exhibits improved homogeneity with respect to Notch activation. This allows a more meaningful evaluation of the functional, cellular and molecular properties of distinct progenitor cell types during normal and abnormal development. The combined functional analysis and gene profiling of the isolated cell types during stage transitions provide a highly valuable resource of stably expressed as well as stage specifically expressed transcriptional regulators, which may be critical for both launching the onset of early NSCs as well as driving their progression through distinct developmental potencies, through Notch activation.

One exciting finding in this study is the more accurate identification of neuroepithelial cells and their properties with respect to Notch activation. Our findings emphasize the ability of enhanced Notch activation to ensure the maintenance of progenitors in a state that allows them to respond to developmental cues. Importantly, high Notch activation does not prevent the progression through distinct fate competences, but rather links the progression through distinct lineages in culture, thus ensuring the execution of the full developmental potential of NE cells. Mechanistically, Notch activation first dictates CNS identity during neural induction. Second, it represses proneural transcriptional activity in NE cells and by that maintains a highly undifferentiated state. Third, Notch active NE cells display augmented expression of cell cycle components, in correlation with maintenance of BrdU incorporation in later derived HES5+ cells. We propose that Notch activation may confer amenability to specification cues mainly by extending the time window during which NE progenitors are exposed to these cues. This model can explain the ability of HES5+ but not HES5− progenitors to undergo complete neuronal specification for various distinct regional identities. This model is supported by *in vivo* studies showing the requirement for successive cell cycles during the specification of both spinal motoneurons and cortical neurons^51^,^52^.

Several intriguing aspects on the molecular forces that drive NSC progression can be drawn from our study. The findings that genes such as *SOX2*, *FOXG1*, *OTX2* and *PAX6* are expressed throughout the culture progression support a model according to which CNS identity is determined during early stages by a core of stably expressed TFs. Nonetheless, the significantly differentially expressed gene sets among stages indicate that stage-specifically expressed genes are also critical for stage transition. We propose that the extensive remodelling capacity of NE cells through progression is provided by stably expressed TFs coacting with consecutively and transiently appearing factors to control NSC progression through Notch activation. It is intriguing to speculate that distinct sets of Notch regulators are consecutively appearing and replacing one another in a relay mechanism to generate potency diversity, while maintaining proliferation capacity through Notch signalling. Such a model should further advance our ability to use these factors to directly induce or maintain specific modules *in vitro*—towards establishing perpetuating NSC types amenable for drug screening, disease modelling and for developing better protocols for deriving specific neuronal and glial lineages.

Our progenitor module dissection approach enables new possibilities of gaining knowledge on progenitor cell dynamics during disease onset and progression. Many disease models, particularly iPS cell based, rely on the ability to generate specific neuronal types suspected to be clinically and physiologically relevant. Our approach offers a unique possibility to specifically isolate damaged or malfunctioning progenitor modules that give rise to the clinically affected neuronal or glial cell types, and to gain deep insights into pathogenic features within such defected modules such as stem cell properties, developmental potential and molecular drivers. Also, the comprehensive array data sets may help to link the expression pattern of disease causing mutated genes along our developmental stage modules with relation to Notch activation. Using our cellular system for deciphering ‘defective units’ during pathogenesis of various nervous system diseases *in vitro* should be a great advancement to the field of disease modelling. Lissencephaly, a developmental cortical disorder, is associated with defects in ‘core’ genes such as *ARX*, stage-specific genes such as *DCX*, and Notch active specific genes such as *VLDLR*. Similarly, Microcephaly is associated with defects in ‘core’ genes such as *MCPH1* and *STIL*, stage-specific genes such as *CENP*, and Notch active specific genes such as *ASPM*. Our data sets may provide insights also to other nervous system disorders such as autism as well as psychiatric disorders. Altered regulation of *DISC1* associated with schizophrenia may be interesting due the fact that expression of this gene appears in culture only starting the M-RG stage. Neurodegenerative diseases associated with mutations or SNPs in genes differentially expressed in our system may also shed light on the potential role of such candidates in predisposition and/or actual elderly onset. For example, we found that *SPON1* and *RRAS2*, overrepresented specifically in L-RG HES5+ cells and thus may relate to gliogenesis, contain SNPs associated with Alzheimer’s disease (*P*=2.07E−4, Odds=15.26). Such findings may imply that the potential embryonic roles of these factors may be inferred also to the malfunction of such SNP-bearing genes during disease onset.

Our system also offers a unique possibility to look into the origin and tumorigenic properties of distinct and yet to be defined brain cancer stem cells. As many of the developmental genes have tumorigenic potential, this study may potentially advance our understanding of how Notch activation is associated with the emergence of distinct brain cancer stem cells. The association of our data sets with brain growth and tumorigenesis also reinvigorates the development of strategies to minimize heterogeneity of progenitors beyond our findings on cortical development. Such studies should also help to develop approaches to control the balance between proliferation and differentiation *in vitro*, to eliminate proliferating progenitors from their differentiated progeny, and to minimize chances of tumorigenicity—towards future implications in preclinical setups.

Last, it will be interesting to test whether the newly described naive PSCs^53^ can be used to generate NE cells and their progeny with employing our described differentiation paradigm, and whether such approach can be helpful to improve harnessing the full neurogenic and gliogenic potential of these cells.

To summarize, because the uniqueness of the data sets and cellular analysis is in their proliferative nature, we envisage that our comprehensive data analyses would serve as a powerful tool to dissect lineage transitions, to identify origin of progenitor cells, to relate them to onset and progression of brain tumours, and to address fundamental questions related to human cortical expansion.

## Methods

### Culturing undifferentiated hESCs

The human ES cell (hESC) line H9 (WA-09, XX, Wicell)-derived BAC transgenic *HES5::eGFP* line^17^ was cultured on mitotically inactivated mouse embryonic fibroblasts (MEFs; Globalstem). Undifferentiated hESCs were maintained in medium containing DMEM/F12, 20% KSR, 1 mM Glutamine, 1% Penicillin/Streptomycin, non-essential amino acids, beta-mercaptoethanol and Fibroblast growth factor 2 (FGF2; 10 ng ml^−1^). Medium was replaced daily and cells were passaged weekly by treating cells with Dispase (6 U ml^−1^, Worthington) followed by mechanical trituration.

### Neural induction and rosette formation and propagation

For neural induction and generation of NE cells, hESC colonies were removed from MEFs by Dispase (6 U ml^−1^, Worthington), dissociated with Accutase (Innovative Cell Technologies, Inc.), plated at subconfluent cell density (40–50 K cells per cm^2^, although twice higher density or alternatively small hESC clusters work well and accelerate confluence) on Matrigel (1:20, BD)-coated dishes, and supplemented with MEF-conditioned media and 10 μM ROCK inhibitor (Y-27632, Tocris) with daily fresh FGF2 (10 ng ml^−1^, R&D). Confluent cultures were subjected to dual SMAD inhibition neural differentiation protocol^54^ containing Noggin (R&D, 250 ng ml^−1^) and SB-431542 (10 μM, Tocris), and further supplemented with LDN-193189 (100 nM, Stemgent; denoted LNSB protocol). HES5::eGFP usually appears on day 8 or 9. To generate E-RG rosettes and subsequent progenitors, NE cells were scrapped from plates on day 10–12, preincubated with Ca^+2^/Mg^+2^ free HBSS followed by collagenase II (2.5 mg ml^−1^), Collagenase IV (2.5 mg ml^−1^) and DNAse (0.5 mg ml^−1^) solution (all from Worthington; 37 °C, 20 min). Cells were then dissociated and replated at high density (500,000 cells per cm^2^) on moist matrigel drops, and grown for additional days till rosettes appeared (E-RG stage). Neural induction and direct formation of E-RG stage rosettes could be also formed by coculture of hESC clusters with MS5 stromal cells as previously described^15^. Briefly, early appearing rosettes on MS5 were harvested mechanically beginning on day 8–10 of differentiation, replated on culture dishes pre-coated with 15 μg ml^−1^ polyornithine (Sigma), 1 μg ml^−1^ Laminin (BD Biosciences) and 1 μg ml^−1^ Fibronectin (BD Biosciences) (Po/Lam/FN) till Day 14, to obtain E-RG rosettes. Under both protocols, early appearing NE cells were cultured from Day 9 with N2 medium (composed of DMEM/F12 and N2 supplement containing Insulin, Apo-transferin, Sodium Selenite, Putrescine and Progesterone), and further supplemented with low SHH (30 ng ml^−1^), FGF8 (100 ng ml^−1^) and BDNF (5 ng ml^−1^). Long-term culture of E-RG rosettes was performed by a weekly mechanical harvesting of rosettes and replating on Po/Lam/FN coated dishes with N2 medium, SHH and FGF8, till day 28. These were replaced by FGF2 (20 ng ml^−1^) and EGF (20 ng ml^−1^) on day 28 (all cytokines from R&D Systems). At each stage cells were either replated as clusters for next passage or subjected to FACS purification by preincubation with Ca^+2^/Mg^+2^-free HBSS followed by mechanical dissociation.

### Neural patterning and cortical laminar specification

Neural patterning was performed in parallel to or immediately following neural induction. For midbrain dopaminergic neuron differentiation, hESCs were neurally induced on matrigel as previously described^7^, and treated with SHH C25II (100 ng ml^−1^, R&D), FGF8 (100 ng ml^−1^) and CHIR99021 (3 μM, Stemgent). On day 12, GFP+ and GFP− NE cells were separated by FACS, replated at very high density (400,000 cells per cm^2^), followed by terminal differentiation with Neurobasal medium (Invitrogen) supplemented with BDNF (20 ng ml^−1^), ascorbic acid (AA; 0.2 mM, Sigma), GDNF (20 ng ml^−1^), TGFβ3 (1 ng ml^−1^), dibutyryl cAMP (0.5 mM, Sigma) and DAPT (10 μM, Tocris) for 14 additional days. For motoneuron differentiation, hESC derived neurally induced cells either on matrigel or MS5 were dissociated on day 12–14, and GFP+ and GFP− cells were separated by FACS and replated on Po/Lam/FN (MS5 protocol) or matrigel drops (matrigel protocol) at medium density (200,000 cells per cm^−2^) and treated with Retinoic Acid (RA, 1 μM, Sigma) and SHH C25II (125 ng ml^−1^) till day 28 as previously described^15^. For early cortical neurons, NE cells on Day 12 were sorted for GFP+ and GFP− populations, replated and cultured with N2 supplemented with AA and BDNF. For inhibition of Notch during terminal differentiation (Fig. 3d), DAPT was added to the differentiation medium (5 μM) from day 2 of differentiation till the rest of differentiation period. For the inhibition of Notch at early neural induction (Supplementary Fig 2b), DAPT was added (5 μM) on day 1 and cells were collected for analysis on day 2, day 6 and day 9.

For neuronal, astroglial or oligodendroglial differentiation of late passages, E-RG rosettes were passaged through mechanical splitting till day 80 or day 220 with FGF2/EGF and BDNF. Either sorted GFP+ and GFP− populations (L-RG stage) or unsorted cells (LNP stage) were replated at high density and differentiated for 14 days in the presence of AA and BDNF for neuronal progeny, with 5% Foetal Bovine Serum (FBS; Invitrogen) for astrocytic progeny, or with AA, BDNF, SHH C25II (100 ng ml^−1^) and FGF8 for oligodendrocytic progeny according to our previous protocol^55^.

### Acute lineage analysis

Neuroectodermal progenitors reaching the NE stage (day 12) using the LNSB/matrigel protocol were separated into HES5+ and HES5− populations, and these were replated, and either immediately fixed and analysed for cell fate/proliferation markers by immunostaining, or maintained for another passage as separate populations till reaching the E-RG stage. Then, these NE derived HES5+ and HES5− cell populations were again separated to newly born HES5+ and HES5− cells, thus creating four distinct lineage related populations. These were either acutely analysed or further maintained till the end of the passage and then again analysed. All analyses were performed 2 h after replating. For BrdU labelling, BrdU (30 μM) was added to cells for 1 h at the second hour following replating, following which cells were subjected to fixation and analysed.

### Immunostaining and confocal imaging

Cells were fixed in 4% paraformaldehyde, 0.15% picric acid, permeabilized and blocked with PBS, 1% bovine serum albumin (BSA), 10% FBS and 0.3% Triton solution, and stained with indicated primary antibodies (see below) followed by Alexa Fluor secondary antibodies (Invitrogen). Cells were imaged in PBS after staining. All cell imaging was carried out in 24-well glass bottom plates (*In Vitro* scientific). Fluorescence images were obtained using a confocal microscope LSM710 (Carl Zeiss MicroImaging, Germany). The confocal and time-lapse images were captured using a 10 × and a 20 × objectives (NA=0.3, 0.8 respectively, Plan-Apochromat). Fluorescence emissions resulting from Ar 488, 543 and 633 nm laser lines for EGFP, CY3 and CY5, respectively, were detected using filter sets supplied by the manufacturer. For DAPI detection we used our mode-locked Ti:Sapphire, fentosecond pulsed, multiphoton laser (Chameleon Ultra II, Coherent, Inc.) at a wavelength of 720 nm. Images and 3D reconstruction movies were generated and analysed using the Zeiss ZEN 2011 software (Carl Zeiss, Inc.) and NIS elements (Nikon). All images were exported in TIF and their contrast and brightness were optimized in Adobe Photoshop under the same settings per each marker across all stages and as well as across HES5+ and HES5− populations.

Neuronal output level quantification was performed by marker/DAPI ratio calculation and statistics of the entire cells in at least two (mostly three) independently taken images for a one representative experiment performed in parallel for all differentiation markers and across all stages. Note that counting is affected also by cells with positive but weak marker appearance, depending on stage examined and epitope tested (such as RELN). For additional quantitative aspects, see also all qPCR charts for all genes across HES5+ and HES5− and across all stages in Supplementary Fig. 4.

### Antibody list

Antibodies for CTIP2 (ab18465, 1:500), CUX1 (ab54583, 1:500), PE-conjugated anti EGFR (ab231, 1:50), LGALS1 (ab25138, 1:1,000), Lin28 (ab46020, 1:1,000), PHH3 (ab5176, 1:250), PLZF (ab104854, 1:100), POU3F2 (ab94977, 1:1,000), SATB2 (ab51502, 1:50), SOX1 (1:1,000), SOX2 (ab79351, 1:500), TBR1 (ab31940, 1:200), TBR2 (ab23345, 1:200) were from Abcam. Antibodies for BrdU (347580, 50 μl per test), KI67 (556003, 1:1,000), phycoerythrin-conjugated (PE) SSEA-3 (560237, 20 μl per test), PE-conjugated F11R (552556; 20 μl per test), Alexa Fluor 647-conjugated TRA-1-60-647 (560850, 5 μl per test), Alexa Fluor 647-conjugated TUJ1 (560340, 1:500) were from BD Biosciences. Antibodies for DCX (AB2253, 1:5,000), O4 (MAB345, 1:25), RELN (MAB5364, 1:200), Tyrosine Hydroxylase (TH, AB152, 1:500) were purchased from Millipore. Antibodies for FABP7 (51010-1-AP, 1:100), S100B (15146-1-AP, 1:100) were from ProteinTech. Antibodies for AP2α (3B5 concentrated, 1:100) and PAX6 (supernatant, 1:16) were from DSHB. Antibody for NESTIN (MO15012, 1:500) was from Neuromics. Antibody for GFAP (Z0334, 1:2,000) was from DAKO. Antibody for β-3-Tubulin (PRB-435P, 1:1,000) was from Covance. Antibody for GLAST (ACSA-1; 130-095-822, 1:10) was from Miltenyi Biotec. Antibody for OTX2 (AF1979; 1:40) was from R&D. Antibody for FOXA2 (SC-6554; 1:100) was from Santa Cruz Biotechnology.

### Quantitative PCR (qPCR) analysis

RNA was extracted using miRNeasy kit (Qiagene) followed by Maxima reverse transcription reaction kit (Fermentas). 1 ng of cDNA was subjected to qPCR using our homemade designed primers (see ‘Primer set list’), ABsoluteQPCR SYBR Green ROX Mix (ABgene) and ViiA-7 cycler (ABI). Threshold cycle values were determined in triplicates and presented as average compared with HPRT. Fold changes were calculated using the 2^−ΔCT^ method. For RT–PCR data evaluation for Fig. 3b,c, RT–PCR data were collected in triplicates, log_2_ transformed and normalized to HPRT. Mean normalized expression values were then normalized to 1 across all differentiation stages of HES5+ and HES5− for each gene separately to reflect the relative expression across stages. Finally, gene expression levels were normalized to 1 for each stage to also reflect the relative abundance of each gene in each stage. The resulting values were grouped into markers for deep layer neurons TBR/RELN and CTIP/FEZF2; and upper layer neurons CUX1/CUX2/SATB2 and displayed in pie charts and bars as shown in Fig. 3b,c, respectively

### Primer set list (all for human genes)

BRN1 (POU3F3) Forward, 5′- TGGACTCAACAGCCACGAC -3′ and Reverse 5′- CTTGAACTGCTTGGCGAAC -3′; BRN2 (POU3F2) Forward, 5′- TGTATGGCAACGTGTTCTCG -3′ and Reverse 5′- CCTCCTCCAACCACTTGTTC -3′; CTIP2 Forward, 5′- TCCAGAGCAATCTCATCGTG -3′ and Reverse 5′- GCATGTGCGTCTTCATGTG -3′; CUX1 Forward, 5′- CAACAAGGAATTTGCTGAAGTG -3′ and Reverse 5′- CTATGGTTTCGGCTTGGTTC -3′; CUX2 Forward, 5′- GAGCTGAGCATCCTGAAAGC -3′ and Reverse 5′- AGGCCTCCTTTGCAATAAGC -3′; EGFR Forward, 5′- GATAGTCGCCCAAAGTTCCGT -3′ and Reverse 5′- CTGAATGACAAGGTAGCGCTG -3′; GFAP Forward, 5′- AGAGATCCGCACGCAGTATG -3′ and Reverse 5′- TCTGCAAACTTGGAGCGGTA -3′; HES5 Forward, 5′- ACCAGCCCAACTCCAAGCT -3′ and Reverse 5′- GGCTTTGCTGTGCTTCAGGTA -3′; HPRT Forward, 5′- TGACACTGGCAAAACAATGCA -3′ and Reverse 5′- GGTCCTTTTCACCAGCAAGCT -3′; PLZF Forward, 5′- CCTTTGTCTGTGATCAGTGCG -3′ and Reverse 5′- CAGTGCCAGTATGGGTCTGC -3′; RELN Forward, 5′- AATGCCGTCACCTTCTGTG -3′ and Reverse 5′- GGAGGACAGAAGCTGTTGTTG -3′; S100β Forward, 5′- GGAAATCAAAGAGCAGGAGGTT -3′ and Reverse 5′- TCCTGGAAGTCACATTCGCC -3′; SATB2 Forward, 5′- TAGCCAAAGAATGCCCTCTC -3′ and Reverse 5′- AAACTCCTGGCACTTGGTTG -3′; TBR1 Forward, 5′- GTCACCGCCTACCAGAACAC -3′ and Reverse 5′- ACAGCCGGTGTAGATCGTG -3′; TBR2 Forward, 5′- AGCCGACAATAACATGCAGGG -3′ and Reverse 5′- TCCTGTCTCATCCAGTGGGA -3′; TH Forward, 5′- CCTCGGATGAGGAAATTGAG -3′ and Reverse 5′- TCTGCTTACACAGCCCGAAC -3′; ENGRAILED1 Forward, 5′-CCCGTGGTCAAAACTGACTC-3′ and Reverse 5′-TTCTTCTTCAGCTTCCTGGTG-3′; FOXA2 Forward, 5′-CCGACTGGAGCAGCTACTATG-3′ and Reverse 5′-TGTACGTGTTCATGCCGTTC-3′; HB9 Forward, 5′-CACCAGTTCAAGCTCAACAAG-3′ and Reverse 5′-TTTTGCTGCGTTTCCATTTC-3′; SOX1 Forward, 5′-GCAAGATGGCCCAGGAGAAC-3′ and Reverse 5′-CGGACATGACCTTCCACTCG-3′; SOX2 Forward, 5′-GCAAGATGGCCCAGGAGAAC-3′ and Reverse 5′-CCGACAAAAGTTTCCACTCGG-3′; NESTIN Forward, 5′-TGGAGGCAAAGAGGGTTCAG-3′ and Reverse 5′-TCGGAGAACTCTGTCCCCAG-3′; PAX6 Forward, 5′-CACACCGGTTTCCTCCTTCA-3′ and Reverse 5′-GGCAGAGCGCTGTAGGTGTTT-3′; TUJ1 Forward, 5′-TGATGAACATGGCATCGAC-3′ and Reverse 5′-TATTTGCCACCTGTGGCTTC-3′; HOXB4 Forward, 5′-CACGGTAAACCCCAATTACG-3′ and Reverse 5′-TCCTTCTCCAGCTCCAAGAC-3′; SIX1 Forward, 5′-TTTAAGAACCGGAGGCAAAG-3′ and Reverse 5′-GGTTCTGCTTGTTGGAGGAG-3′; FEZF2 Forward, 5′-CCCAGGAAAAGCCACATAAATG-3′ and Reverse 5′-GGATGCGGATATGCGTGTT-3′; NR2F1 Forward, 5′-AGAAGCTCAAGGCGCTACAC-3′ and Reverse 5′-GACTTCTCCTGCAGGCTCTC-3′; PCP4 Forward, 5′-GGTGCATCCATGTCAATGTC-3′ and Reverse 5′-GCAACCAATGGAAAAGACAAG-3′; FABP7 Forward, 5′-GGTGGAGGCTTTCTGTGCTACC-3′ and Reverse 5′-AAGCCCACGCCTAGAGCCTT-3′; GLAST Forward, 5′-CCCTTGGGTTTTTATTGGAGG-3′ and Reverse 5′-ATGGGTAGGGTGGCAGAACT-3′; LIN28A Forward, 5′-ACAGGTGCTACAACTGTGGAGG-3′ and Reverse 5′-AGAAGTGGCACTTCTTGGGC-3′; LGR5 Forward, 5′-GGAAATCATGCCTTACAGAGC-3′ and Reverse 5′-ACACTCCAAATGCACAGCAC-3′; DCN Forward, 5′-AATGCCATCTTCGAGTGGTC-3′ and Reverse 5′-AGAGTTGTGTCAGGGGGAAG-3′; LGALS1 Forward, 5′-AGCCTGGAAGTGTTGCAGAG-3′ and Reverse 5′-TGGGGAACTTGAATTCGTATC-3′; EZH2 Forward, 5′-CAGCCTTGTGACAGTTCGTG-3′ and Reverse 5′-GGAAAGCGGTTTTGACACTC-3′; NR2E1 Forward, 5′-AGACCAGCTGATGCTTTTGG-3′ and Reverse 5′-GTTAGCATCAACCGGAATGG-3′.

### Fluorescent-activated cell sorting (FACS)

Cell sorting was performed using ARIA flow cytometer (Beckton Dickinson). NE cells were dissociated with collagenase II (2.5 mg ml^−1^), Collagenase IV (2.5 mg ml^−1^) and DNAse (10 mg ml^−1^; all from Worthington) solution (37C, 20 min). E-RG and subsequent stages were dissociated either with Accutase (37 °C, 15 min) or Ca^+2^/Mg^+2^ free HBSS (RT, 1 h). All stages were FACS sorted to GFP+ and GFP−-gated populations following exclusion of dead cells with DAPI. L-RG and LNP stages were also analysed for EGFR abundance. Undifferentiated hESCs were sorted for the pluripotency markers Tra-1-60 and SSEA-3.

### Microarray data processing and analysis

GeneChip PrimeView Human Arrays were used for all array hybridizations. Normalized log_2_ transformed probe level intensities were collapsed onto MGI gene symbols yielding 19,448 gene level measurements. Next, genes were filtered for a minimum log_2_ change of one or greater across between any pair of samples as well as a minimum log_2_ expression level of three or greater in at least one sample. The results yielded 6,371 gene entries, which are listed in Supplementary Data 1.

### Stage-wise clustering

To get a high-resolution view of the underlying dynamics and evaluate the distinct expression patterns, we performed clustering *k*-means (*k*=100; *n*=26) on the time series of the positive samples based on a set of eight predefined patterns. The expression patterns were defined based on all possibilities of gene upregulation between consecutive differentiation stages, for example, upregulated from hESCs to NE, but down in E-RG, upregulated from hESCs to NE and not changing from NE to E-RG but downregulated from E-RG to M-RG and so on. Differential expression between two stages was defined as a minimum log_2_ expression change of one or greater. In total, we classified 495 genes, which are listed in Supplementary Data 4, to follow one of these patterns. Subsequently, each cluster was subjected to gene set enrichment analysis. The results are shown in Fig. 6a.

### Notch active specific genes

Genes expressed in a stage-specific manner for HES5+ with respect to the HES5− populations and vice versa were determined by first clustering all time points using *k*-means (*k*=100). Subsequently, hESC expression levels were subtracted from each stage. Next, HES5+ expression levels were divided by HES5- and vice versa. Next, we selected clusters that at the same time point (i) showed an average fold change exceeding 1.4 in one of the time points; and (ii) average fold changes that are <1.2 in all remaining time points. Results are reported in Supplementary Data 2 and 3, respectively and in Fig. 4a (for genes highly expressed in HES5+), or referred within text (for genes highly expressed in HES5−).

### Gene enrichment analysis

Stage-specific obtained gene data sets were analysed for enriched categories using Ingenuity Pathway Analysis (IPA) and selected resulting categories were plotted as heatmap (Fig. 6b), or directly referred to within text.

## Author contributions

Y.E. conceived and designed the experimental paradigm. R.E. and Y.E. developed the cell culture isolation techniques. R.E. and Y.Y. performed ES cell culture and consecutive cell derivation and propagation. R.E. performed long-term propagation experiments with the help of Y.Y., performed neural patterning experiments, performed and analysed most qPCRs and performed immunostainings together with Y.Y.. Y.Y. performed confocal imaging and analysis for all experiments, including confocal 3D reconstruction analyses. Y.Y., N.M. and R.V. performed and analysed acute lineage analysis experiments. M.J.Z. performed microarray data processing and analysis, and qPCR analysis and presentation. E.D and I.G.V performed microarray data processing and normalization, stage-wise clustering and gene enrichment analysis. J.J.H and G.R. performed the microarray assays and initial processing. H.M. and C.L. contributed to the qPCR experiments and analysis. R.E., Y.Y., M.J.Z. and Y.E. interpreted the data. Y.Y. and Y.E. generated all figures and wrote the manuscript. M.J.Z. and A.M. contributed to critical reading of the manuscript.

## Additional information

**Accession codes:** All microarray data generated in this study have been deposited in the NCBI Gene Expression Omnibus under accession code GSE65369.

**How to cite this article:** Edri, R. *et al*. Analysing human neural stem cell ontogeny by consecutive isolation of Notch active neural progenitors. *Nat. Commun.* 6:6500 doi: 10.1038/ncomms7500 (2015).

## Supplementary Material

Supplementary Figures Supplementary Figures 1-7

Supplementary Data 1 Global gene expression arrays for HES5+ and HES5- populations during NSC progression. Gene expression intensities for ES cells as well as HES5+ and HES5- cells during 220 days of propagation *in vitro*. Normalized and collapsed log2 transformed probe level intensities are shown.

Supplementary Data 2 HES5+ vs. HES5- expression intensity ratios during NSC progression. Genes whose expression is overrepresented in HES5+ cells compared to HES5- cells at the different stages are shown. The intensity level of each gene in ES cells was subtracted from all values prior to ratio calculation.

Supplementary Data 3 HES5- vs. HES5+ expression intensity ratios during NSC progression. Genes whose expression is overrepresented in HES5- cells compared to HES5+ cells at the different stages are shown. The intensity level of each gene in ES cells was subtracted from all values prior to ratio calculation.

Supplementary Data 4 Global gene expression cluster analysis for stage specifically expressed genes. Selected trends of global gene expression clustering comprising 496 genes.

Supplementary Movie 1 Three-dimensional reconstruction analysis of E-RG rosettes structure composition. Confocal z-sections of HES5::eGFP (green), combined with immunostaining for PAX6 (red) and DAPI staining for nuclei (blue) are shown. Image stacks with 17-19 planes per stack, at a spacing of 2 μm, and frame averaging of four images per plane were collected. All fluorescence images were confocal images of optical slice thickness ~0.9 μm. Scale bar: 50 μm.

Supplementary Movie 2 Three-dimensional reconstruction analysis of M-RG rosettes structure composition. Confocal z-sections of HES5::eGFP (green), combined with immunostaining for PAX6 (red) and DAPI staining for nuclei (blue) are shown. Image stacks with 17-19 planes per stack, at a spacing of 2 μm, and frame averaging of four images per plane were collected. All fluorescence images were confocal images of optical slice thickness ~0.9 μm. Scale bar: 50 μm.
