# Supplementary material for: Analysing human neural stem cell ontogeny by consecutive isolation of Notch active neural progenitors
Source: Nat Commun. 2015 Mar 23;6:6500. doi: 10.1038/ncomms7500 (PMC4383005; doi:10.1038/ncomms7500)
Supplement: Supplementary Figures — 1-7 [file ncomms7500-s1.pdf]

Supplementary Figure 1

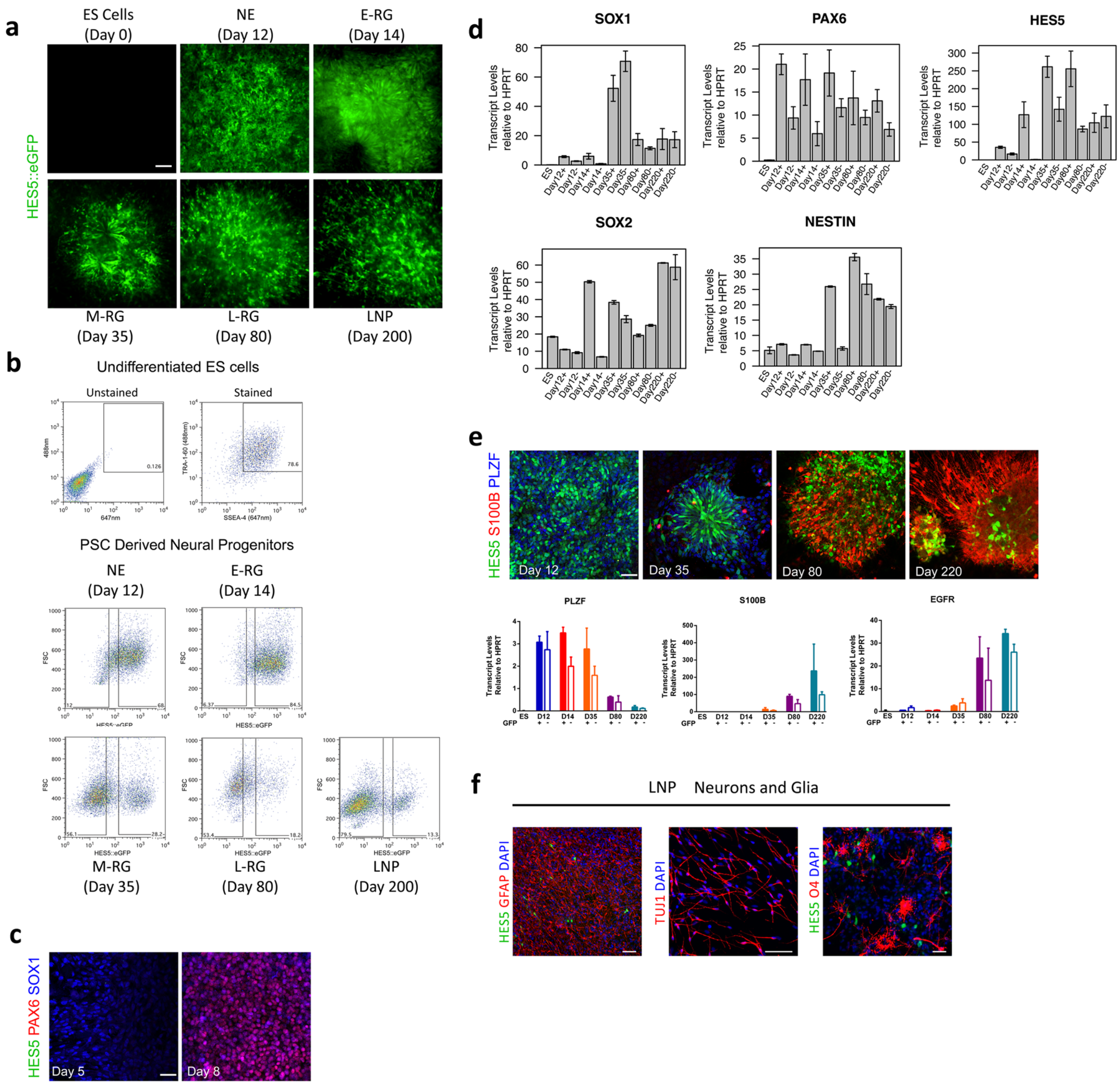

Supplementary Figure 1. HES5 expression dynamics in PSC derived neural progenitors.
(a) Fluorescent microscopy of *HES5::eGFP* during long-term differentiation shows dynamic morphological features through neural progenitor cell progression in vitro. Scale bar: 50μm**. (b)** Top: FACS charts depicting ES cells purified for pluripotency markers. SSEA-4 and TRA-1-60 surface markers are presented (right). Unstained cells are shown on the left. Bottom: *HES5::eGFP* percentages for all stages are shown. Percentages indicated are representative of three independent experiments. **(c)** SOX1 and PAX6 expression in neuroectodermal cells at days 5 and 8, prior onset of *HES5::eGFP*. Scale bar: 25μm**. (d)** Quantitative PCR analysis of transcript levels of neural stem and progenitor cell markers (for whom immunostainings is shown in **Fig. 1c).** Relative expression levels for HES5+ and HES5- samples across the entire progression period are shown. Values were obtained from three technical replicates. Statistical analysis: mean ± SEM. **(e)** Top: Expression dynamics of early rosette markers (PLZF) and late glial progenitor markers (S100B) during progression in vitro are shown. Scale bar: 50μm. Bottom: qPCR analysis of PLZF, S100B and EGFR are shown. **(f)** Combined *HES5::eGFP* reporter intensity and immunostainings for multipotency marker expression of progeny derived from LNP progenitor stages. GFAP, TUJ1 and O4 are shown from left to right. Scale bar: 50μm

Supplementary Figure 2

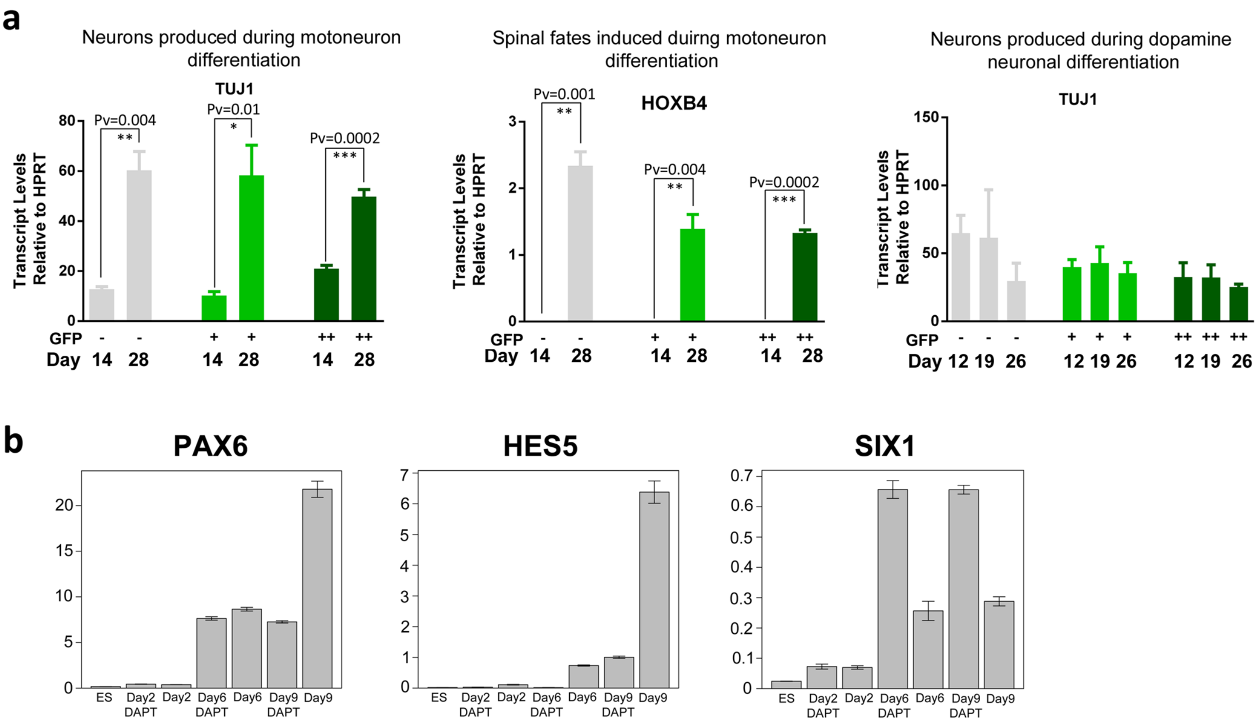

Supplementary Figure 2. CNS fate specification and regional patterning potential in HES5+ and HES5- progenitors.

(a) Quantitative PCR analysis of transcript levels of the regional marker HOXB4 and the neuronal marker TUJ1 during motoneuron differentiation (left column), or TUJ1 expression during dopamine neuron differentiation (right column). High (++, dark green bars), medium (+, light green bars) and low (-, gray bars) HES5 expressing progenitors, in their proliferative state (Day12 or Day14) and following terminal neuronal differentiation (Day 28 for motoneurons, Day19 and Day26 for dopamine neurons) are shown. All transcript levels shown are normalized to respective HPRT levels in each sample. Values were obtained from three technical replicates. Statistical analysis: mean ± SEM; T-Test: (***) $P < 0.001$; (**) $P < 0.01$; (*) $P < 0.05$. **(b)** CNS fate specification required Notch activation. Neurally induced cells were treated with or without the Notch inhibitor DAPT on either day 2 or day 6 of neural induction and harvested for qPCR analysis on Day 9. Relative expression (compared to HPRT) of early appearing CNS markers PAX6 and HES5 and the neural crest/placodal marker SIX1 on Day 9 is shown. Values were obtained from three technical replicates. Statistical analysis: mean ± SEM.

Supplementary Figure 3

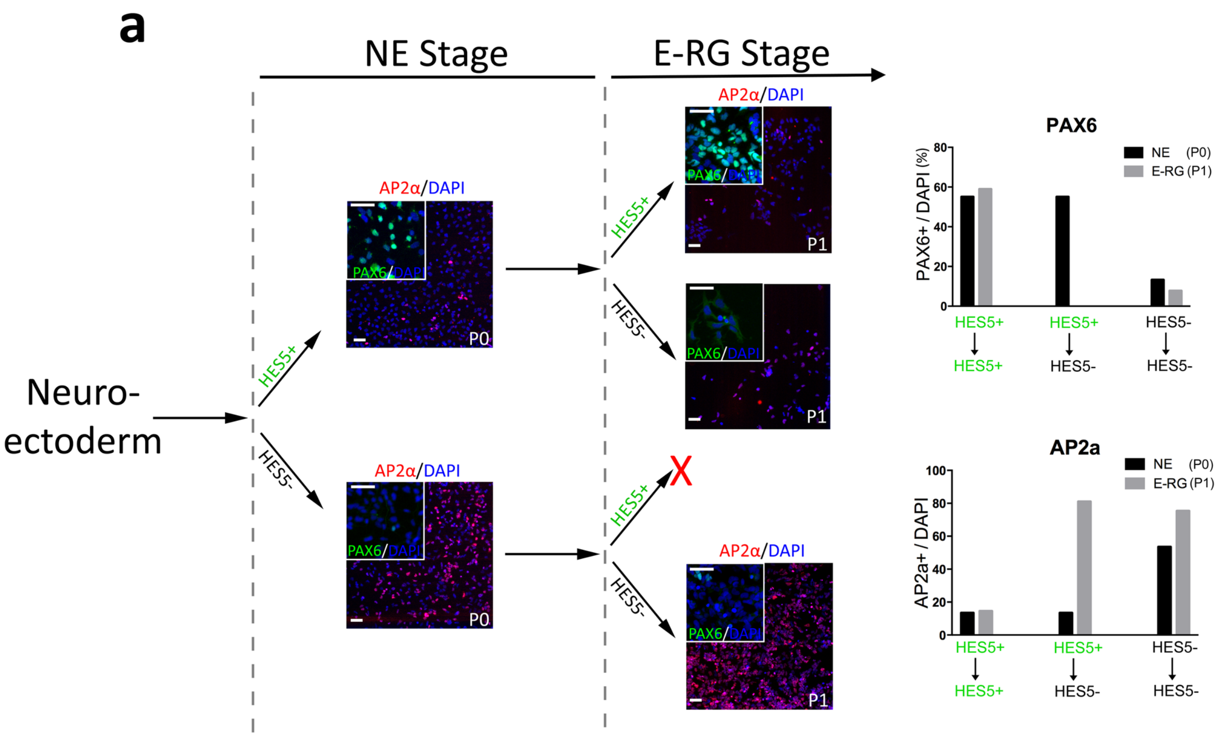

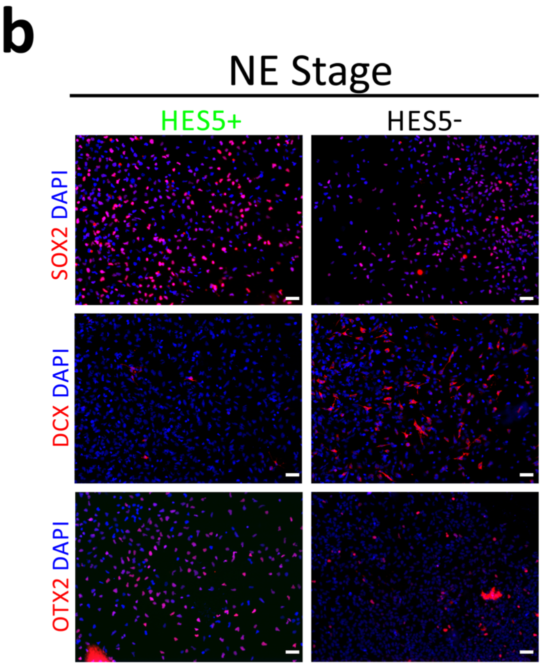

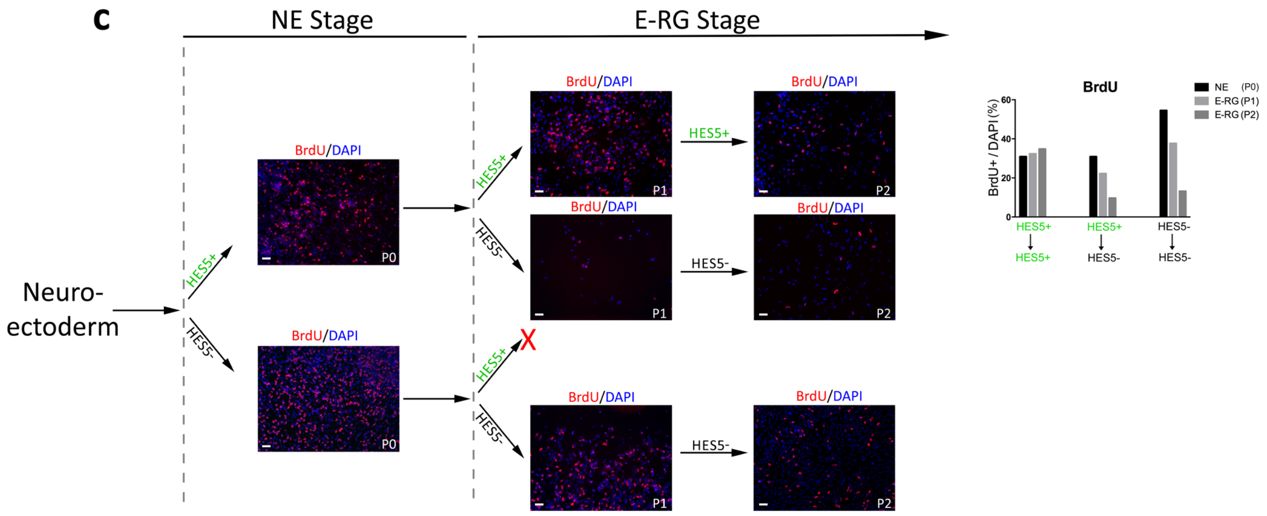

Supplementary Figure 3. Cell fate and proliferation marker segregation in consecutively sorted HES5+ and HES5- cells.

(a) Immunostainings for CNS and non-CNS markers PAX6 and AP2a, respectively, in NE stage HES5+ and HES5- cells and their directly and consecutively derived E-RG stage HES5+ and HES5- progeny (see Method for details). Acute fixation and staining following sorting is shown. Quantification of PAX6 and AP2a cell ratios reflecting segregation of CNS and non-CNS cell fates via Notch activation and inactivation, respectively, is shown. Lineage relations for each stage analyzed (NE, E-RG) are indicated by vertical arrows on the x-axis. Scale bar for images and insets: 50µm**.** **(b)** Additional anterior CNS and NSC markers OTX2 and SOX2, as well as the neuronal marker DCX are shown for NE stage HES5+ and HES5- progenitors acutely sorted and analyzed. Scale bar: 50µm. **(c)** Immunostainings for the S-Phase marker BrdU are shown in an experiment performed similar to the one presented in **a**. Immunostainings were performed immediately after sorting, re-plating, and 1 hour of BrdU labeling are shown. Quantification of BrdU+ cell ratios is shown through stages examined on the right. Quantifications in **a** through **c** are representative of at least 2 independent experiments. Scale bar: 25µm**.**

Supplementary Figure 4

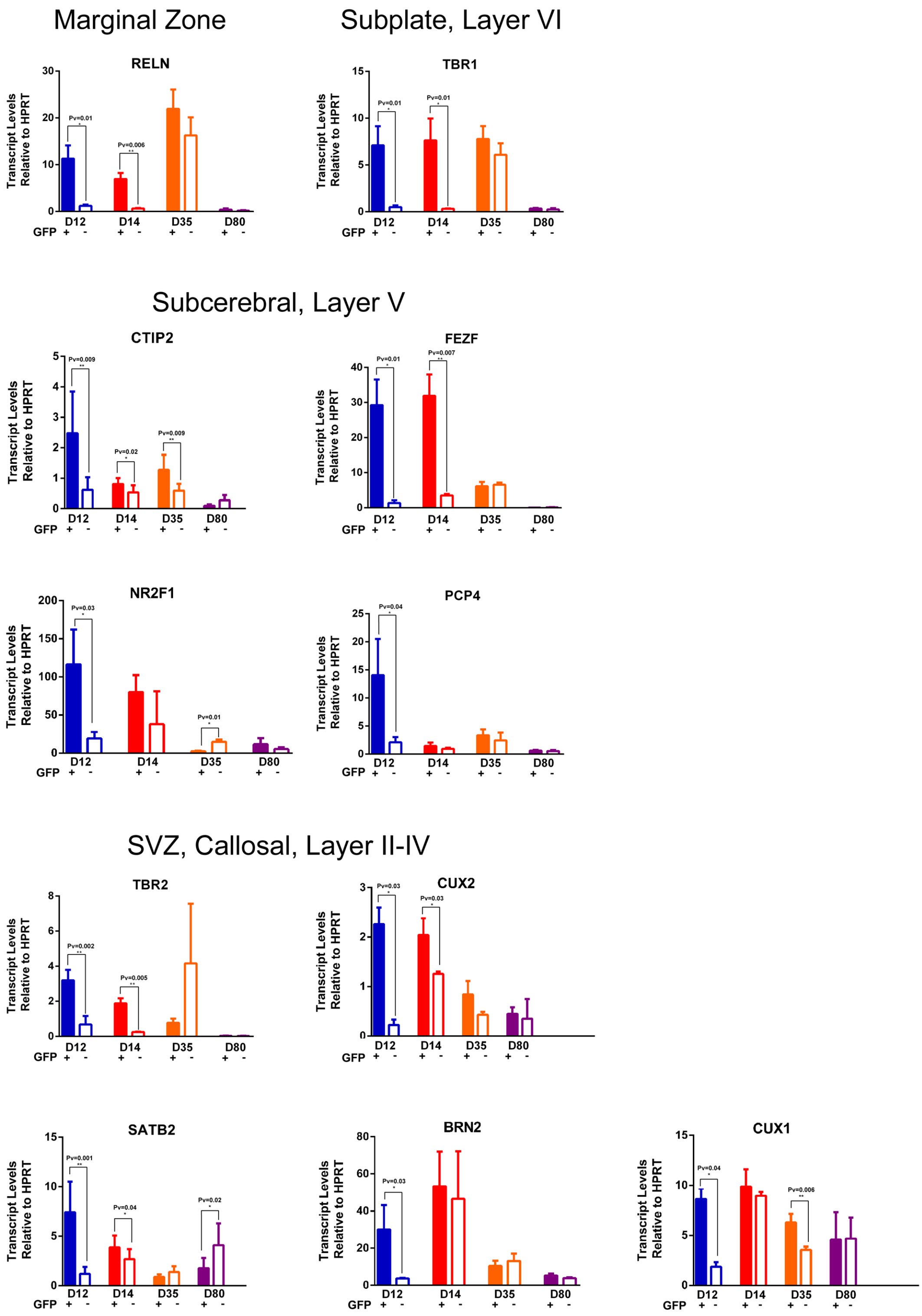

Supplementary Figure 4. Transcript validation of cortical lamination by PSC derived consecutively appearing neural progenitors. Individual qPCR analyses of laminar markers in neuronal progeny derived from HES5+ and HES5- progenitor populations from NE, E-RG, M-RG and L-RG stages. Values from selected genes were collapsed together for generating the pie charts and bars shown in **Figure 3c and 3d,** respectively. All transcript levels shown are normalized to respective HPRT levels in each sample. Values shown were obtained from three technical replicates of a representative experiment. Statistical analysis: mean ± SEM; T-Test: (***) $\boldsymbol{P} < 0.001$; (**) $\boldsymbol{P} < 0.01$; (*) $\boldsymbol{P} < 0.05$.

Supplementary Figure 5

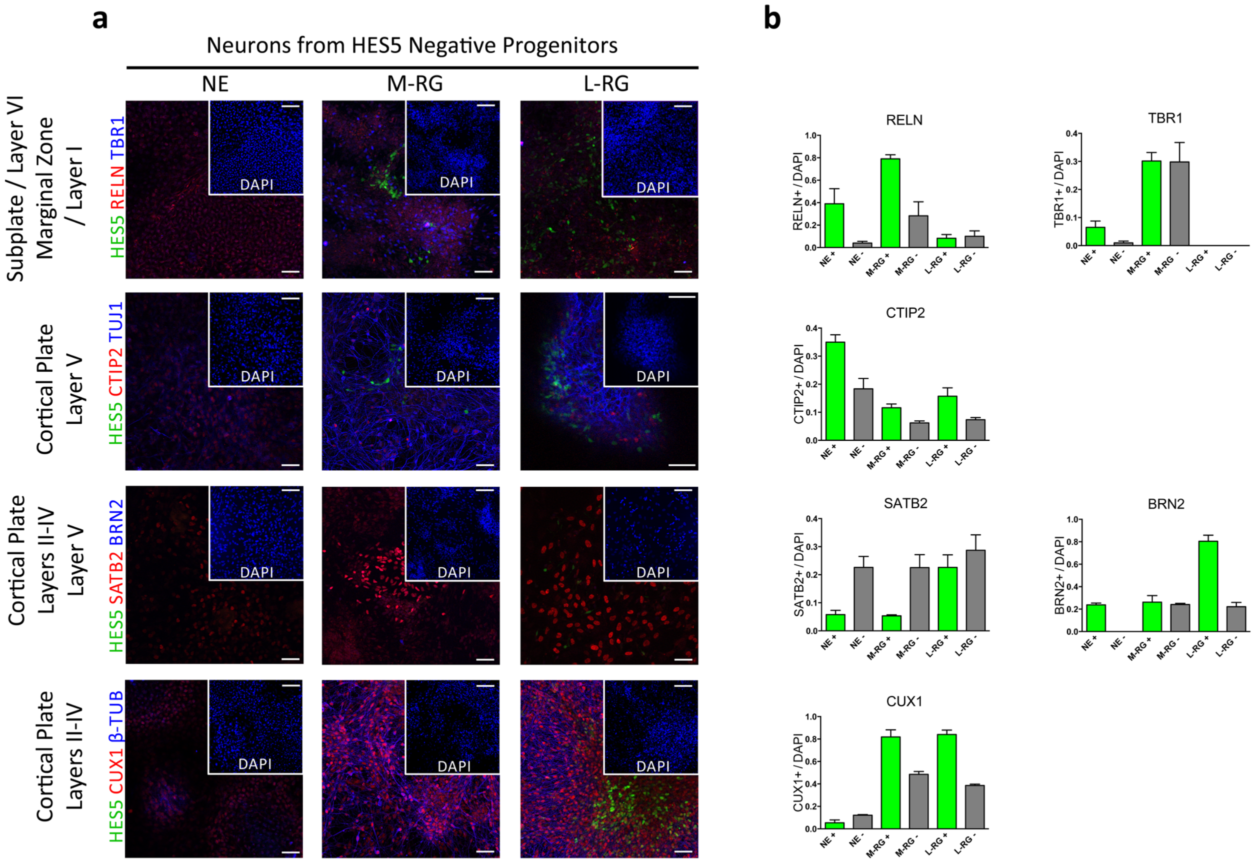

Supplementary Figure 5. Differentiation capacity of HES5- progenitor cell stages.
(a) Combined *HES5::eGFP* reporter expression and immunostainings of cortical layer specific neuronal markers for neuronal progeny derived from HES5- cells across stages is shown. Panels and stainings are ordered identically to the ones shown for HES5+ progenitor stages in **Fig. 3a** (See details in figure legend). Insets show compressed magnification of a matched DAPI image for the entire corresponding image. Scale bars: 50µm for images, 100µm for Insets. **(b)** Quantification of marker immunofluorescence intensity of the neuronal progeny shown in **a.** Entire image cell counting relative to DAPI of at least 2 independently taken images for one representative experiment is shown. Also shown in each of the charts is the quantification of cell ratios expressing these specific neuronal markers from HES5+ progenitors (for which images are shown in **Fig. 3a**). Statistical analysis: mean ± SEM.

Supplementary Figure 6

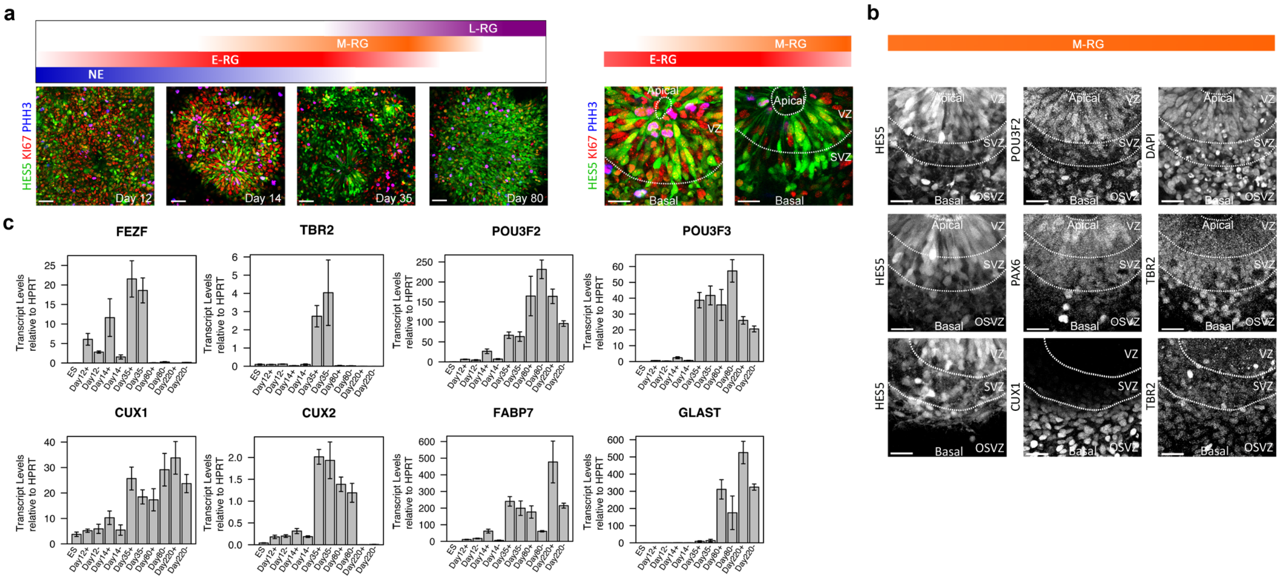

Supplementary Figure 6. Spatiotemporal progenitor marker expression during progression in vitro: rosettes as VZ and SVZ equivalents.
(a) Combined *HES5::eGFP* reporter expression and immunostainings for the mitotic (M-Phase) marker PHH3 and the cell cycle marker KI67. Scale bar: 50μm. Right panel shows high power magnification of E-RG and M-RG rosettes shown on the left. Scale bar: 2μm. **(b)** Separate channel presentation for high power magnification images of E-RG and M-RG rosettes shown in **Fig. 4d**. POU3F2, TBR2, and CUX1 are shown. Dashed lines demarcate proposed VZ, SVZ and OSVZ regions, containing apical RG, INPs and putative basal RG, respectively. Scale bar: 25μm. **(c)** Quantitative PCR validations of transcript levels for all VZ and SVZ markers whose gene array levels are represented by heatmaps on **Figure 4b** are shown. Relative expression levels for HES5+ and HES5- samples across the entire progression period are shown. Values were obtained from three technical replicates of a representative experiment. Statistical analysis: mean ± SEM.

Supplementary Figure 7

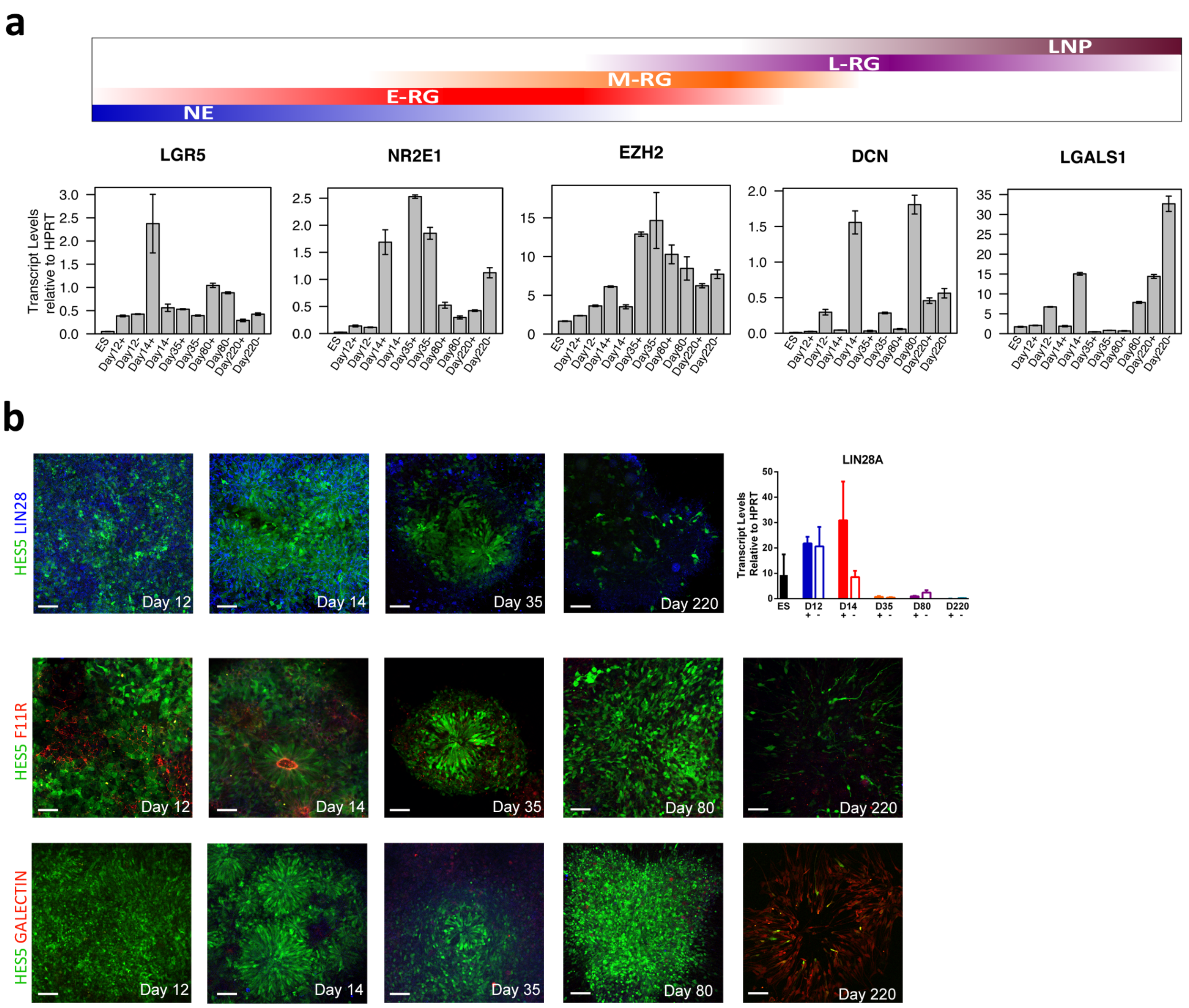

Supplementary Figure 7. Stage specific marker validation.
(a) qPCR validation of transcript levels for selected markers across all stages and across HES5+ and HES5- populations. Relative expression (compared to HPRT) is shown. Values were obtained from three technical replicates. Statistical analysis: mean ± SEM. **(b)** Combined *HES5::eGFP* reporter expression and immunostainings for selected stage specific identified markers is shown. Scale bar: 50µm. Statistical analysis for qPCR on top right pnael: mean ± SEM.
